# The enhanced energy metabolism in the tumor margin mediated by RRAD promotes the progression of oral squamous cell carcinoma

**DOI:** 10.1038/s41419-024-06759-7

**Published:** 2024-05-29

**Authors:** Aoming Cheng, Qiaoshi Xu, Bo Li, Lirui Zhang, Hao Wang, Chang Liu, Zhengxue Han, Zhien Feng

**Affiliations:** Department of Oral and Maxillofacial-Head and Neck Oncology, Beijing Stomatological Hospital, Capital Medical University, Beijing, China

**Keywords:** Cancer metabolism, Oral cancer, Prognostic markers

## Abstract

The tumor margin as the invasive front has been proven to be closely related to the progression and metastasis of oral squamous cell carcinoma (OSCC). However, how tumor cells in the marginal region obtain the extra energy needed for tumor progression is still unknown. Here, we used spatial metabolomics and the spatial transcriptome to identify enhanced energy metabolism in the tumor margin of OSCC and identified that the downregulation of Ras-related glycolysis inhibitor and calcium channel regulator (RRAD) in tumor cells mediated this process. The absence of RRAD enhanced the ingestion of glucose and malignant behaviors of tumor cells both in vivo and in vitro. Mechanically, the downregulation of RRAD promoted the internal flow of Ca^2+^ and elevated its concentration in the nucleus, which resulted in the activation of the CAMKIV-CREB1 axis to induce the transcription of the glucose transporter GLUT3. GLUT inhibitor-1, as an inhibitor of GLUT3, could suppress this vigorous energy metabolism and malignant behaviors caused by the downregulation of RRAD. Taken together, our study revealed that enhanced energy metabolism in the tumor margin mediated by RRAD promotes the progression of OSCC and proved that GLUT3 is a potential target for future treatment of OSCC.

## Introduction

Oral squamous cell carcinoma (OSCC), a common malignant tumor in the head and neck, has a poor prognosis accompanied by cervical lymph node metastasis [1]. Metabolic reprogramming and inner spatial heterogeneity play important roles during malignant progression for most malignant tumors, including OSCC [2]. Research has increasingly shown that the metabolic phenotypes of cells in different inner sublocations of tumors are spatially heterogeneous to help them compete for resources and adapt for success in their microenvironment [3]. The aggressiveness of cells in the invasive front has a close connection to tumor development and progression. Compared to the relatively static cells in the tumor center, the cells in the dynamic invasive front have higher growth rates and preferential expansion but also face more challenges due to the complex tumor microenvironment [4]. This phenomenon leads to increased energy consumption. Specifically, initiating invasion through the surrounding basement membrane and extracellular matrix (ECM) is an energy-intensive step for cells in the tumor margin [5]. To meet this demand, leader cells in the invasive front will enhance the energy status to respond to the higher metabolic costs for ECM remodeling while moving forward compared to the cells behind the tumor center [6, 7]. However, whether similar leader cells with strong metabolism exist in the tumor margin of OSCC and how these cells obtain extra energy are still unknown.

As the main energy-supplying and most cost-effective substance, glucose is the first choice for cancer cells as metabolic fuel. Regulation of glucose transporters (GLUTs) is a critical requirement for cell metabolism. The upregulation of GLUT1 and GLUT3 to enhance glycolytic flux has been reported in numerous cancer types [8–12]. In addition to the common functions compared with GLUT1, GLUT3 shows a much higher affinity for glucose [13]. GLUT3 tends to be expressed in cells located in glucose-deficient environments that still have substantial demands for glucose, such as in brain tissue [14]. A previous study also indicated that as a migration leader, marginal cancer cells increased glucose uptake and promoted energy status by enhancing aerobic glycolysis. Given the similar large energy requirement in the two situations, whether GLUT3 participates in promoting energy status in the tumor margin still needs further exploration.

In this study, we performed spatial metabolomics and spatial transcriptome analysis to explore the inner metabolic heterogeneity of OSCC, which indicated that the tumor margin has a hypermetabolic status. We also demonstrated that low Ras-related glycolysis inhibitor and calcium channel regulator (RRAD) expression in the tumor margin activated glucose metabolism. Mechanistically, low expression of RRAD leads to increased intracellular and nuclear Ca^2+^ concentrations and activates the Calcium/Calmodulin Dependent Protein Kinase IV(CAMKIV)-CAMP Responsive Element Binding Protein 1(CREB1) axis, resulting in the upregulation of GLUT3. Furthermore, GLUT inhibitor-1 could significantly counteract the vigorous metabolic level and cellular proliferation, migration, and invasion of OSCC cells mediated by GLUT3 upregulation in vitro and in vivo. These results indicated that RRAD and GLUT3 act as key factors to improve the metabolic status of peripheral cells and promote the progression of OSCC. Moreover, limiting tumor glucose uptake by targeting GLUT3 with oral inhibitors may become a promising therapy for OSCC.

## Results

### Spatially resolved multiomics reveals metabolic heterogeneity of tumor margins in OSCC

**Fig. 1:**
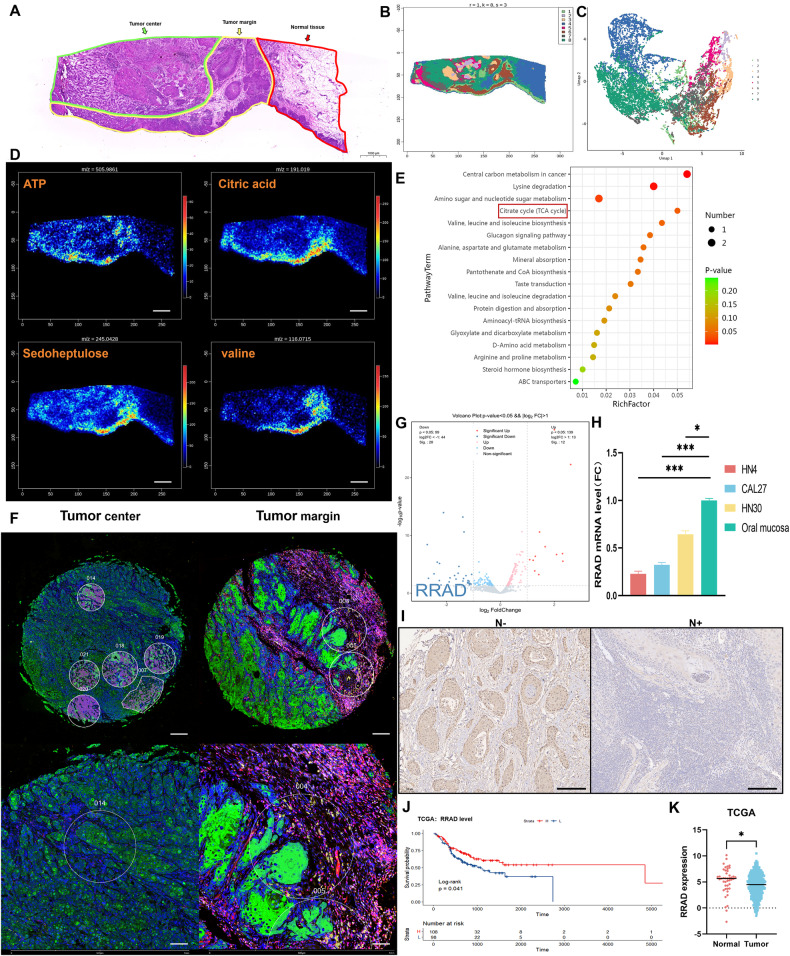
Energy status of tumor margin in OSCC is upregulation and correlates with low expression of RRAD. **A** H&E stain image of patient with advanced OSCC, divided into different areas guided by pathology experts. Scale bar: 1000 μm. **B** Visual cluster map of global metabolite expression levels. **C** Uniform Manifold Approximation and Projection for Dimension Reduction (UMAP) of different clusters. **D** MS images of representative energy-related metabolites in OSCC tissues (intensity in color scale is relative value). Scale bar: 500 μm. **E** KEGG pathway enrichment analysis based on differential metabolites in tumor margin. **F** DSP analysis was performed in FFPEs of OSCC patients, ROIs were separately selected from tumor center and tumor margin. Scale bars: 500 μm and 100 μm. **G** Volcano Plot of DEGs between tumor center and margin, RRAD was downregulated in tumor margin. **H** Relative RRAD mRNA expression was detected in OSCC cell lines, normal oral mucosa. *P < 0.05, ***P < 0.001. **I** Representative IHC images of RRAD correlation with tumor status. Scale bar: 200 μm. **J** Survival analysis was performed in the OSCC dataset from the TCGA database. The correlation between RRAD expression and overall survival. **K** Relative RRAD mRNA expression in tumor and normal tissue based on OSCC dataset from the TCGA database. *P < 0.05.

To investigate the internal metabolic heterogeneity of OSCC and explore the potential influence of different sublocations during malignant progression, we chose a fresh frozen surgical specimen from a patient with advanced OSCC for spatial metabolomics based on the AFADESI-mass spectrometry imaging (MSI) platform. After removal of necrotic tissue, the histological regions were divided into three types, tumor center, tumor margin and normal tissue, under pathologic expert guidance (Fig. 1A). To further display variations in metabolite concentrations of different subregions, we performed clustering analysis based on the global level of metabolites. Analysis of clusters and histological partition revealed that cluster 6 overlapped the main area of the tumor margin, which indicated that compared with the other two regions, the tumor margin showed a substantial difference in metabolic characteristics (Fig. 1B, C). Subsequently, analysis of MS images showed that ATP was highly concentrated in the tumor margin as well as other core products of various energy supply pathways, such as citric acid and sedoheptulose (Fig. 1D). Under the guidance of the Kyoto Encyclopedia of Genes and Genomes (KEGG) enrichment of metabolic pathways, we found that the characteristic metabolites of the tumor margin were mainly enriched in energy supply pathways, including the TCA cycle (Fig. 1E). All the results above indicated that as the leader in the invasive front, the cells in the tumor margin had altered bioenergetic characteristics to meet the energy requirements for invasion and migration compared with those in the tumor center. To further explore key factors that may promote the energy status at the invasive front, we next selected formalin-fixed, paraffin-embedded (FFPE) samples of primary lesions derived from four patients with OSCC, including two patients with lymph node metastases, and performed spatial transcriptome profiling based on the NanoString Digital Spatial Profiling (DSP) instrument. Then, regions of interest (ROIs) were selected in both the tumor center and margin for further differential analysis (Fig. 1F). Finally, the differentially expressed genes (DEGs) included 40 genes, 28 of which were downregulated in the tumor margin (Fig. 1G and Supplementary Table 1). Among the DEGs, RRAD seemed to be correlated with energy metabolism, and the current research does not support a potential regulatory relationship or interaction between RRAD and other proteins in the DEGs of tumor margin. A previous study suggested that RRAD can inhibit Ca_v_1.2, a subtype of L-type voltage-gated Ca^2+^ channel (VGCC), and mediate the cellular Ca^2+^ concentration [15–17]. Some cancer studies have also indicated that RRAD may influence energy utilization as an antioncogene [18–21]. To determine the expression of RRAD in OSCC, we examined the mRNA level of RRAD in OSCC cell lines, as well as oral mucosa. Three OSCC cell lines had a lower level of RRAD than normal oral epithelial cells (Fig. 1H). Immunohistochemical (IHC) staining in OSCC tissue suggested that patients with cervical lymph metastases expressed RRAD at low levels (Fig. 1I). The Cancer Genome Atlas (TCGA) dataset shows that the prognosis of patients with low RRAD mRNA levels is much poorer than that of patients with high RRAD mRNA levels, and the RRAD mRNA level of tumors is also lower than that of normal tissue, consistent with our results (Fig. 1J, K). These data indicate that downregulation of RRAD correlated with malignant progression in OSCC patients.

### Inhibition of RRAD expression enhances energy metabolic levels and promotes malignant progression in OSCC cells

**Fig. 2:**
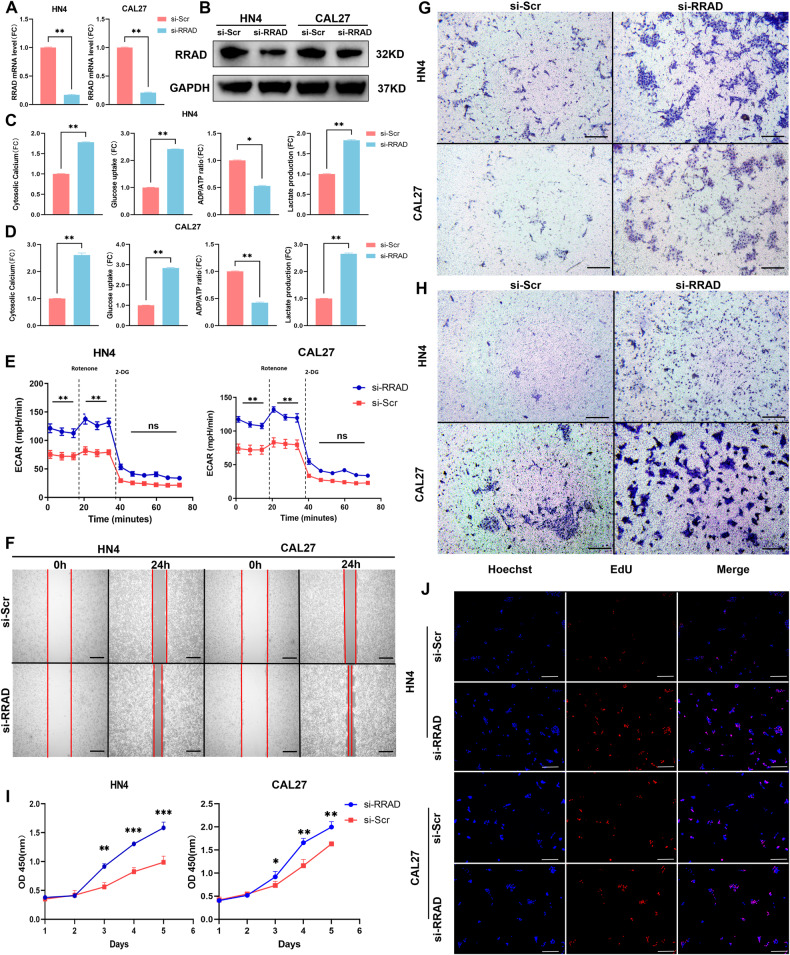
Low expression of RRAD promotes energy status and malignant progression in OSCC cell lines. **A**, **B** Transfection efficiency was detected using PCR and Western blot after RRAD-specific siRNA transfection for 48 h in HN4 and CAL27 cells. **P < 0.01. **C**, **D** Intracellular Ca^2+^ concentration, glucose uptake ability, lactate production and ADP:ATP ratio were detected after silencing RRAD in HN4 and CAL27 cell lines. *P < 0.05, **P < 0.01. **E** The real-time ECAR was analyzed by the Seahorse method after silencing RRAD. **P < 0.01. **F** A wound-healing assay was conducted to detect the migration ability of OSCC cells after silencing RRAD. Scale bar: 250 µm. **G**, **H** Migration and invasion abilities were studied using a Transwell assay after silencing RRAD in HN4 and CAL27 cells. Scale bar: 250 µm. **I**, **J** The CCK-8 and EdU assays were performed to detect proliferation ability after silencing RRAD in HN4 and CAL27 cells. Scale bar: 250 µm, *P < 0.05, **P < 0.01, ***P < 0.001.

**Fig. 3:**
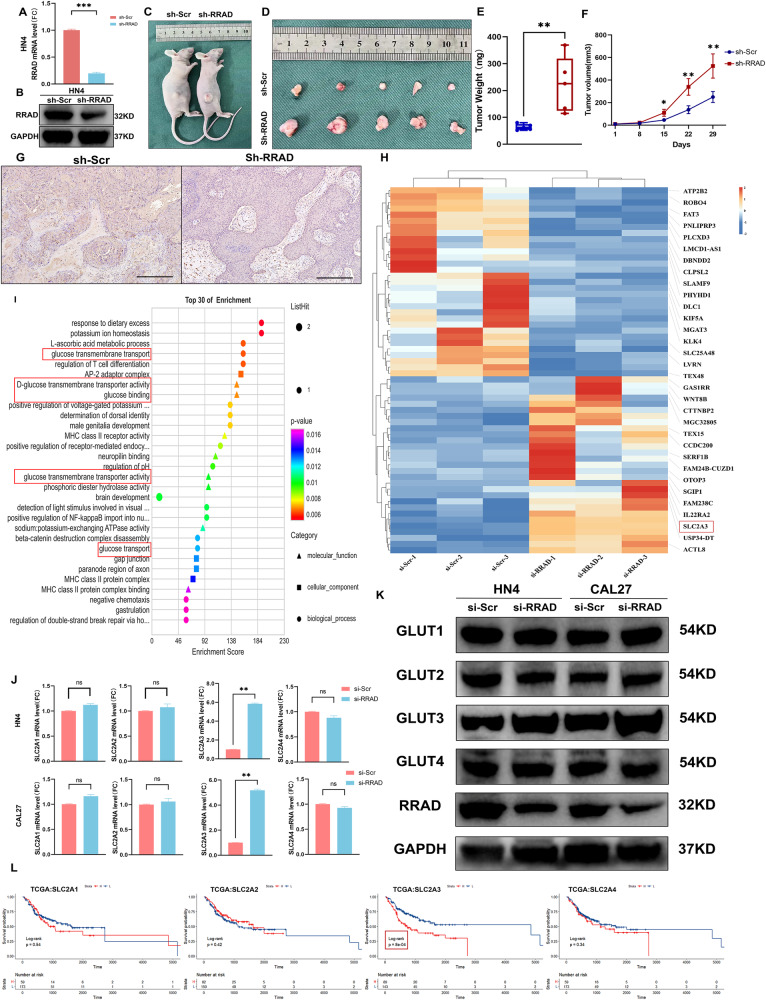
GLUT3 is key downstream of low expression of RRAD. **A**, **B** Transfection efficiency was detected using PCR and Western blot after RRAD-specific shRNA transfection in HN4 cells. ***P < 0.001. **C**, **D** Representative images of tumors derived from the xenograft model were shown. **E** Tumor weight was measured after excision from mice. **P < 0.01. **F** The tumor volume was measured and analyzed once a week. *P < 0.05, **P < 0.01. **G** IHC staining for RRAD was conducted on xenograft tumors. Scale bar: 200 μm. **H** The heatmap of DEGs of si-Scr vs. si-RRAD. **I** The GO enrichment based on DEGs of si-Scr vs. si-RRAD. **J** The relative SLC2A1, SLC2A2, SLC2A3, and SLC2A4 mRNA levels of HN4 and CAL27 cell lines. **P < 0.01. **K** GLUT1, GLUT2, GLUT3, and GLUT4 were detected after silencing RRAD in HN4 and CAL27 cell lines. **L** Survival analysis was performed in the OSCC dataset from the TCGA database. The correlation between SLC2A1, SLC2A2, SLC2A3, and SLC2A4 expression and overall survival.

To further explore the biological mechanism underlying the spatial differential expression of RRAD in OSCC, we established silencing models in CAL27 and HN4 cell lines because of their low expression levels (Fig. 2A, B and Supplementary Fig. 1), and a series of experiments were performed in vitro. As mentioned above, the capacity to inhibit Ca^2+^ channels in RRAD may influence malignant progression in OSCC cells by regulating the intracellular Ca^2+^ concentration. A Fluo-4 AM assay showed that the increased intracellular Ca^2+^ concentration was associated with the silencing of RRAD expression. Furthermore, a 2-NBDG assay revealed that without the limitation of RRAD, OSCC cell lines showed higher affinity and greater transport capacity in glucose uptake. Similar results were also presented in the lactate assay, after 24 h cultured, the lactate concentration in spent media of HN4 and CAL27 cell lines were increased rapidly after silencing RRAD. The ADP:ATP ratio shows the most intuitionistic result, knocking down RRAD leads to a lower ADP:ATP ratio (Fig. 2C, D), which means that silencing of RRAD in OSCC cell lines promoted ATP production and utilization efficiency compared to that of the control group. We also implemented real-time extracellular acidification rate (ECAR) analysis using the Seahorse platform. Glycolysis and glycolytic capacity were significantly increased after RRAD silencing in the HN4 and CAL27 cell lines (Fig. 2E). The results above indicate that silencing RRAD enhanced the energy metabolic level in OSCC cells. In addition, the wound-healing assay revealed that after silencing RRAD, HN4, and CAL27 cells migrated faster than the control group (Fig. 2F and Supplementary Fig. 2A). The number of migrating and invading HN4 and CAL27 cells was significantly increased after RRAD knockdown without or with Matrigel (Fig. 2G, H and Supplementary Fig. 2B, C). CCK-8 and EdU assays revealed that silencing RRAD also increased cell proliferation and DNA replication compared to those of the control group (Fig. 2I, J and Supplementary Fig. 2D). The results of in vivo studies confirmed the conclusions above. Lentiviral knockdown of RRAD was performed on HN4 and the effect was verified by PCR and Western blot prior to establishing the animal model (Fig. 3A, B and Supplementary Fig. 3A). Tumors with silenced RRAD grew faster than their counterparts in the control group (Fig. 3C–G). These results demonstrated that for OSCC cells in the tumor margin, low expression of RRAD and promotion of intracellular Ca^2+^ concentration acted as key factors to improve energy metabolic levels and in turn promoted malignant progression, although the mechanism needs further investigation.

### GLUT3 is a key downstream target of RRAD silencing

To investigate the impact of silencing RRAD on changes in potential downstream gene transcription related to energy metabolic promotion, we performed RNA-seq transcriptomic analysis of the RRAD-silenced OSCC cells (Fig. 3H). Gene Ontology (GO) pathway enrichment analysis of DEGs revealed that significantly enriched terms were strongly correlated with glucose transmembrane transport, D-glucose transmembrane transporter activity and glucose binding (Fig. 3I). Analysis of DEGs showed that the expression level of SLC2A3, the coding gene of GLUT3, was increased significantly (FC = 12.06, *P* < 0.001). Next, we tested whether the expression of other common GLUTs was changed. The results revealed that GLUT3, but not GLUT1, GLUT2, and GLUT4, was significantly upregulated at both the mRNA and protein levels after silencing RRAD (Fig. 3J, K and Supplementary Fig. 3B–F). Furthermore, TCGA data were used to assess the association between the transcription of their genes and the overall survival of patients with OSCC. The Kaplan–Meier (KM) analysis indicated that only upregulation of SLC2A3 was correlated with poor survival outcome, which indicates that among GLUTs, GLUT3 played a more significant role in OSCC (Fig. 3L). As the key downstream target of RRAD silencing, GLUT3 serves as a bridge connecting the intracellular Ca^2+^ concentration and energy metabolic level and may become a potential therapeutic target for OSCC.

### Nuclear Ca^2+^ activates CAMKIV and induces GLUT3 expression in OSCC cells via CREB1

**Fig. 4:**
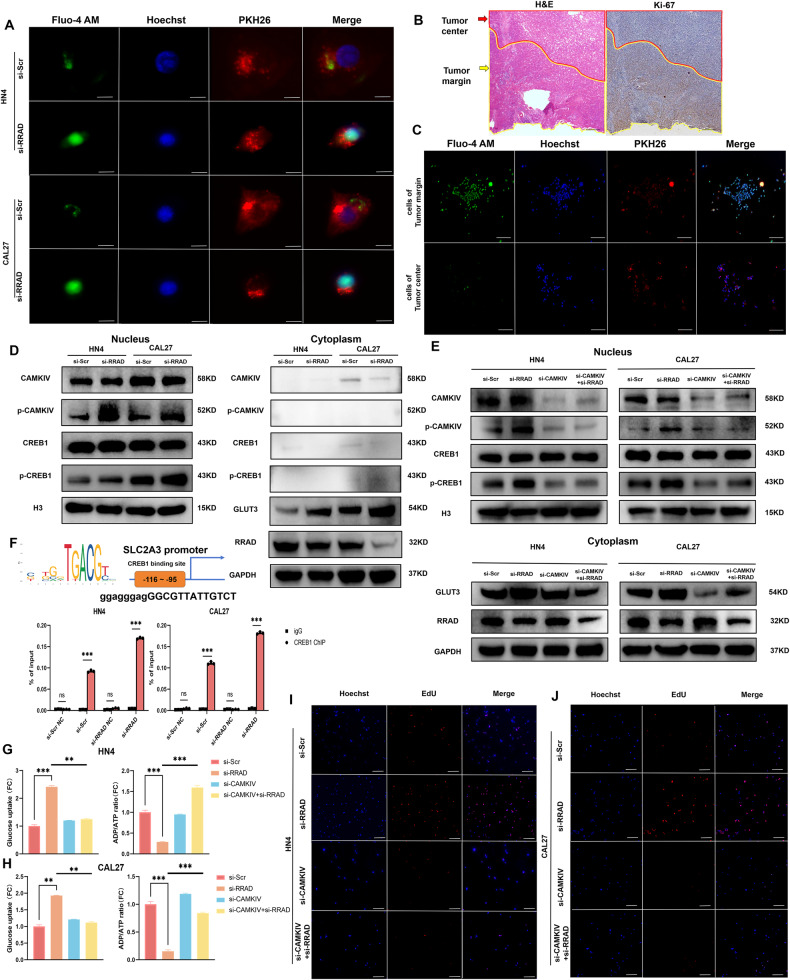
The low expression of RRAD increased nuclear Ca^2+^ level and upregulated GLUT3 by activating the CAMKIV-CREB1 axis. **A** Intracellular and nuclear Ca^2+^ distribution after silencing RRAD in OSCC cells. Scale bar: 50 μm. **B** H&E and IHC stain images of patients with advanced OSCC, dividing into different areas guided by pathology experts for further cell separation. Scale bar: 500 μm. **C** Intracellular Ca^2+^ concentration in OSCC cells derived from tumor center and tumor margin. Scale bar: 250 μm. **D** CAMKIV, p-CAMKIV, CREB1, and p-CREB1 were respectively detected in nuclear and cytoplasm, RRAD, GLUT3 in cytoplasm were detected with western blot after silencing RRAD. **E** CAMKIV, p-CAMKIV, CREB1, and p-CREB1 were detected in nuclear and RRAD, GLUT3 in cytoplasm was detected with Western blot after RRAD-specific and CAMKIV-specific siRNA transfection. **F** ChIP-qPCR analysis of CREB1 binding on the known site in the SLC2A3 promoter region in HN4 and CAL27 cells. ***P < 0.001. **G**, **H** Glucose uptake ability and ADP:ATP ratio were measured after RRAD-specific and CAMKIV-specific siRNA transfection in OSCC cells. **P < 0.01, ***P < 0.001. **I**, **J** EdU assay was performed to detect proliferation ability after RRAD-specific and CAMKIV-specific siRNA transfection. Scale bar: 250 μm.

Previous studies identified the CREB1-binding site in the promoter region of SLC2A3 and showed that CREB1 could modulate GLUT3 expression [22, 23]. CREB1 is the best-known transcription factor that is activated by elevated Ca^2+^ levels. However, how a rise in intracellular Ca^2+^ influences the nucleus is unclear. Considering that the structure of the nuclear envelope allows relatively free diffusion of Ca^2+^ from the cytoplasm, we hypothesized that after silencing RRAD, the increase in the cytoplasmic Ca^2+^ concentration enhances nuclear Ca^2+^ levels and leads to nuclear Ca^2+^ relocalization, which then activates downstream transcription factors and upregulates GLUT3. The results revealed that the green fluorescence showed a significant enhancement and mostly covered blue fluorescence after silencing of RRAD compared to that of the control group, which indicated that the nuclear Ca^2+^ concentration increased (Fig. 4A). To further verify the above result in the patient sample, under the guidance of pathologists, we segmented the tumor margin and tumor center of a fresh primary lesion sample from a patient with advanced OSCC, separately extracted tumor cells from different regions and measured Ca^2+^ concentration. Previous studies indicated that tumor margin shows strong positive intensity of Ki-67 immunoreaction [3]. Corresponding to H&E staining of the frozen section, the IHC staining showed that Ki-67 was also highly expressed in the tumor margin (Fig. 4B). In addition, tumor cells from the tumor margin show higher Ca^2+^ concentration compared to those from tumor center (Fig. 4C). CREB1 is mainly activated through phosphorylation induced by CAMKs. CAMKIV, a subtype of CAMKs highly concentrated within the nucleus, plays a vital role in glucose conditioning according to previous studies [24, 25]. Previous findings have indicated that CAMKIV is a likely candidate to link both sides. Subsequently, we determined the subcellular location and phosphorylation state of CAMKIV, and the results indicated that CAMKIV was mostly expressed in the nucleus and that increasing the nuclear Ca^2+^ concentration induced its phosphorylation after silencing RRAD. We also demonstrated enhanced phosphorylation of CREB1 at Ser133 in the nucleus, which indicated that CREB1 was transcriptionally activated and upregulated GLUT3 (Fig. 4D and Supplementary Fig. 4A–F). To further prove the function of CAMKIV in this process, we knocked down CAMKIV with small interfering RNA (siRNA). Western blotting also showed that after inhibiting CAMKIV, the expression of p-CREB1 and GLUT3 was decreased, while the expression of RRAD and CREB1 was not affected (Fig. 4E and Supplementary Fig. 5A–F). Previous studies claimed that CREB1 could initiate the transcription of GLUT3 by binding to its promoter region [22, 23]. To testify this conclusion, chromatin immunoprecipitation (ChIP) assays were performed and the results suggested that CREB1 could bind to the site (−116 to −95 bp) in GLUT3 promoter region, with a significantly higher level of binding in the si-RRAD group (Fig. 4F). In addition, the promotion of glucose uptake and energy levels by increasing nuclear Ca^2+^ concentration was significantly inhibited by blocking the CAMKIV-CREB1 axis (Fig. 4G, H), and similar results were also found for the DNA replication capacity (Fig. 4I, J and Supplementary Fig. 6A). Collectively, these findings show that the nuclear Ca^2+^ concentration induced GLUT3 expression in OSCC cells by activating the CAMKIV-CREB1 axis and consequently enhanced the capacity of these cells to promote their energy level and respond to higher metabolic costs for invasion as leader cells in the tumor margin.

### GLUT inhibitor-1 impedes tumor progression of OSCC

**Fig. 5:**
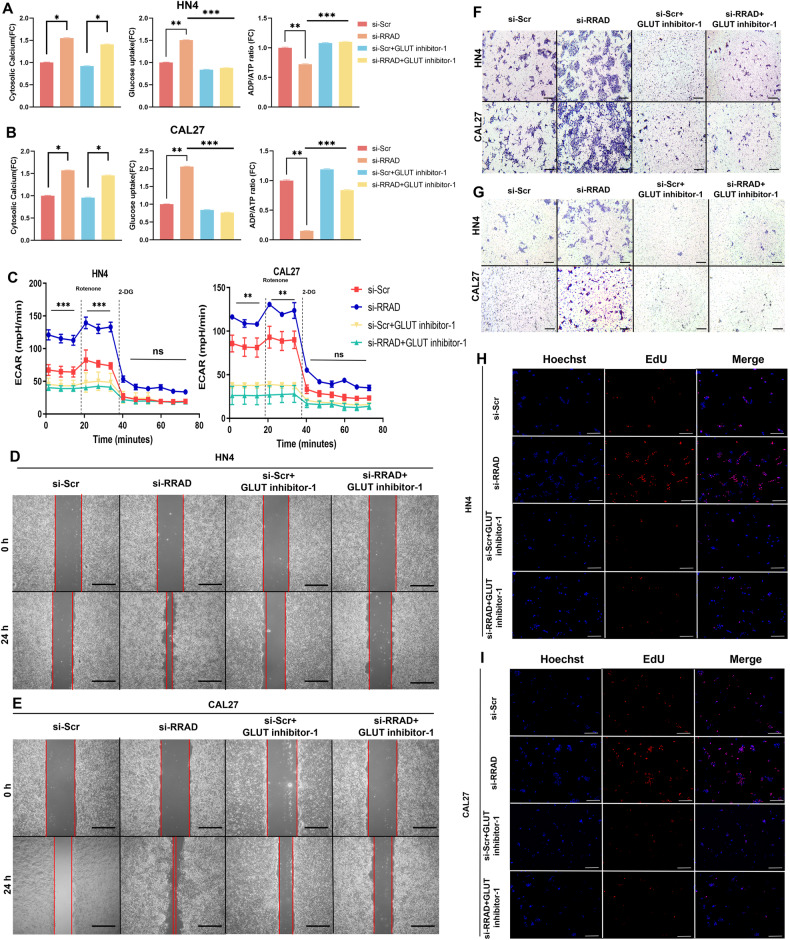
GLUT inhibitor-1 counteracted the vigorous metabolic level and malignant progression of OSCC cells mediated by GLUT3 upregulation. **A**, **B** Intracellular Ca^2+^ concentration, glucose uptake ability and ADP:ATP ratio were detected in OSCC cells treated with 200 nM GLUT inhibitor-1 for 24 h. *P < 0.05, **P < 0.01, ***P < 0.001. **C** The real-time ECAR was analyzed by the Seahorse method in OSCC cells treated with 200 nM GLUT inhibitor-1. **P < 0.01, ***P < 0.001. **D**, **E** A wound-healing assay was performed to detect the migration ability of OSCC cells treated with 200 nM GLUT inhibitor-1. Scale bar: 250 μm. **F**, **G** Migration and invasion abilities were studied using a Transwell assay after treating with 200 nM GLUT inhibitor-1. Scale bar: 250 μm. **H**, **I** EdU assay was performed to detect proliferation ability after treating with 200 nM GLUT inhibitor-1 Scale bar: 250 μm.

**Fig. 6:**
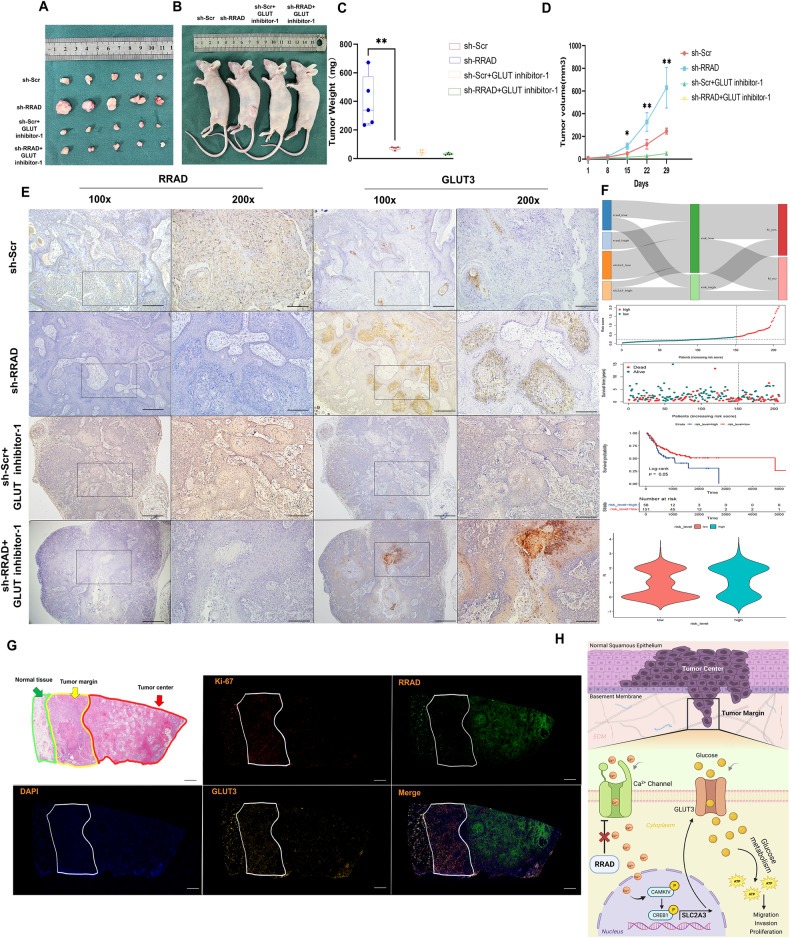
RRAD expression negatively correlated with GLUT3 in OSCC tissues. **A**, **B** Representative images of tumors derived from the xenograft model were shown. **C** Tumor weight was measured after excision from mice. **P < 0.01. **D** The tumor volume was measured and analyzed once a week. *P < 0.05, ** *P* < 0.01. **E** IHC staining for RRAD and GLUT3 was conducted on xenograft tumors. Scale bar: 500 μm and 250 μm. **F** Construction of risk model based on OSCC dataset from the TCGA database. Survival analysis based on RRAD and GLUT3 mRNA level. And the condition of lymph node metastases of patients with different risk levels. **G** H&E stain and multiplex immunofluorescence of the primary lesion tissue, including tumor margin and tumor center. Scale bar: 1000 μm. **H** A schematic diagram shows that low expression of RRAD in the tumor margin increased intracellular and nuclear Ca^2+^ concentration and upregulated GLUT3 by activating CAMKIV-CREB1 axis, which improved the energy status of OSCC cells in the tumor margin to lead progression.

To further explore whether GLUT3-mediated glucose utilization is needed for malignant progression and the feasibility of GLUT3 inhibition as a potential therapy for OSCC patients, we treated HN4 and CAL27 cells with the small molecule GLUT inhibitor, GLUT inhibitor-1. Among the reported GLUT inhibitors, GLUT inhibitor-1 is an inhibitor that only targets GLUT1 and GLUT3 and has little influence on other GLUTs according to previous reports [26]. Given the lack of a specific inhibitor for GLUT3 and the expression level of GLUT1 showing no difference in whether RRAD is silenced in OSCC cells (Fig. 3J–K), GLUT inhibitor-1 seems to be the best choice. As the results showed, after the application of GLUT inhibitor-1, the promotion of glucose uptake caused by silencing RRAD was counteracted, and similar results were also displayed in the ADP:ATP ratio, which indicated that the enhanced energy status was markedly suppressed, while the intracellular Ca^2+^ concentration was not influenced (Fig. 5A, B). ECAR analysis showed that the glycolytic capacity of HN4 and CAL27 cells was significantly decreased by GLUT inhibitor-1 (Fig. 5C). Furthermore, the results of the wound assay, migration assay, and invasion assay illustrated that the migration and invasion mediated by silencing RRAD were significantly inhibited by GLUT inhibitor-1 without or with Matrigel in OSCC cells (Fig. 5D–G and Supplementary Fig. 6B–D), as well as the DNA replication capacity (Fig. 5H, I and Supplementary Fig. 6E). All the results above indicated that inhibiting GLUTs could efficiently block the malignant progression of OSCC cells caused by the upregulation of GLUT3 in vitro. Subsequently, to confirm the in vitro results, we added GLUT inhibitor-1 to the drinking water of mice in safe proportions to investigate the effect of oral administration. Similar to the in vitro study, tumors with RRAD silencing grew fastest among all four groups, while the groups treated orally with GLUT inhibitor-1 showed tumor growth inhibition. This trend started at week 2 and was observed throughout the whole process (Fig. 6A–D). These results implied that inhibition of GLUT3 and further inhibition of the high metabolic level of OSCC cells in the tumor margin is a potential emerging therapeutic strategy.

### RRAD expression is negatively correlated with GLUT3 in OSCC tissues and indicates poor prognosis

Based on all the results above, we confirmed that RRAD could significantly influence the expression of GLUT3 and related with malignant progression. These findings were confirmed by IHC staining of tumor tissue from nude mice and indicated that GLUT inhibitor-1 did not influence the expression of GLUT3 (Fig. 6E). Furthermore, a risk prediction model was built based on TCGA data, and the results indicated that high-risk patients with low expression of RRAD and high expression of GLUT3 simultaneously had a poorer prognosis and a higher rate of lymph node metastases (Fig. 6F). We then performed a multiplex immunofluorescence assay on the primary lesion sample from patient with advanced OSCC, and the result revealed that a similar low expression of RRAD and increased expression of GLUT3 simultaneously existed at the tumor margin, as supported by the above findings. In addition, Ki-67 was also found to be highly expressed at the tumor margin, indicating increased metabolic rate and cellular proliferation associated with the lower expression of RRAD (Fig. 6G). Furthermore, the Tissue Microarrays (TMA) included 135 tumor tissues from patients were used to determine the relationship between RRAD and GLUT3. The results showed a similar tendency (*r* = −0.82, *P* < 0.01), and patients with lymph node metastases also showed lower expression of RRAD along with higher expression levels of GLUT3 (Supplementary Fig. 7A, B).

These data illustrated that low expression of RRAD increased the nuclear Ca^2+^ concentration, activated the CAMKIV-CREB1 axis, and resulted in upregulated GLUT3, which is the reason behind the high metabolic status of the tumor margin. This process is manifested as the low expression of RRAD in peripheral cells, resulting in an enhancement in energy status to guide malignant progression as leader cells and causing poor outcomes in OSCC patients (Fig. 6H).

## Discussion

For OSCC patients, cervical lymph node metastases result in poor prognosis. The 5-year overall survival (OS) rate of patients with traditional treatments is still approximately 50% [27]. Therefore, the development of new therapeutic options and methods is urgently needed. With the assistance of spatial multiomics, we found that OSCC cells located in the tumor margin have a more active energy status to support them as leaders during malignant progression. Furthermore, the low expression of RRAD in the tumor margin resulted in increased GLUT3, which is the basis of vigorous metabolism. Moreover, limiting glucose uptake during the disordered energy status of leader cells and then hindering the malignant progression of OSCC may become a potential metabolic intervention for current therapy.

While faced with multiple pressures, including energy production, invasion, and migration, different subgroups of cells in tumors serve as predominantly ‘specialists’ for different tasks, and it is optimal for particular subgroups to be specially placed in the best position for their task. For instance, in breast tumors, cells in the tumor margin upregulated CAXII, caspase-3, and other markers of invasion and proliferation compared to their counterparts in the tumor center [28]. Ki-67 staining suggests that proliferating cells at the tumor edge outnumber cells at the core, and similar patterns have also been noted in cultured tumor spheroids and organoids, including OSCC [3, 29–32]. All the evidence above indicates that the physical location within the tumor is another additional important determinant of cell progression. Specifically, cancer cells located at the periphery are more connected with malignant progression. To support energy requirements for leading migration, cells concentrate on energy production by localizing mitochondria to the invasive front or increasing glycolysis to respond to fluctuating energetic demands [5, 6]. With the assistance of spatial metabolomics and spatial transcriptomics, we found that the metabolism of the tumor margin in OSCC was more active, and the cells in the tumor margin produced more ATP to address the higher metabolic costs for invasion. Our study also demonstrated that the energy gap between the two regions was rooted in the difference in the intracellular Ca^2+^ concentration caused by the downregulation of RRAD.

The role of RRAD in tumors requires further investigation, and increasing evidence has shown that low expression of RRAD in tumors promotes energy metabolism during malignant progression [33]. Furthermore, the significant inhibition of RRAD on Ca^2+^ channels indicated that the level of RRAD influences the intracellular Ca^2+^ concentration [15], which may influence the nuclear Ca^2+^ concentration considering that the nuclear envelope allows relatively free diffusion of Ca^2+^ according to a previous study [34]. However, the link between Ca^2+^ signaling changed by RRAD levels and metabolic reprogramming is still unclear. In our study, we demonstrated that peripheral cancer cells promoted energy status by decreasing the expression of RRAD. The current study demonstrated that RRAD suppressed aerobic glycolysis by influencing GLUT1 and GLUT4 in other solid tumors [18, 20, 21]. For the transcriptional regulation of RRAD, some studies indicated that hypermethylation in RRAD promoter region is associated with reduced RRAD expression in tumor tissues and leading to poor prognosis [35–37]. Wang et al. found that activation of the Ras pathway upregulates the expression of DNA methyltransferases (DNMTs), and Ras-responsive transcription factors (RRTF) may recruit DNMTs to the RRAD promoter to induce DNA methylation [38]. Other studies also suggested that the downregulation of RRAD may result from transcription factor inactivation. RRAD is a direct transcriptional target of p53. Jennis M et al demonstrated that the expression level of RRAD decreases when p53 is deficient [39], however, the study about the transcriptional regulation of RRAD in OSCC is still lacking. Our study confirmed that low expression of RRAD influenced the nuclear Ca^2+^ concentration. Regulating GLUT3 via the CAMKIV-CREB1 axis is a new biological function of RRAD that links the Ca^2+^-related signaling pathway and metabolic reprogramming during the malignant progression of OSCC. Furthermore, our study first demonstrated that OSCC cells located in the tumor margin promote glucose uptake to support energy costs, leading to malignant progression through low expression levels of RRAD, but the reasons for this change and upstream key factors still need further investigation.

A high capability of glucose uptake and utilization is the common choice for most tumors [40]. GLUT3 has already been well-studied in other tumors. However, in OSCC, this molecule needs further exploration. Our experiments proved that upregulation of GLUT3 in the tumor margin is essential for acquiring a high capability of glucose uptake and then supporting energy consumption for leading-edge invasion and migration. Interestingly, according to our spatial multiomics results, GLUT1, as a universal GLUT, did not show internal spatial expression differences, and we speculated that it may be correlated with potential glucose competition with other cells in complex boundary environments, which is more suitable for the strong affinity of GLUT3 for glucose. Furthermore, significant spatial differences in GLUT3 expression in OSCC yield a potential therapeutic opportunity. In our study, we demonstrated that GLUT inhibitor-1, an oral small molecule GLUT inhibitor, efficiently inhibited the malignant progression of OSCC in vitro and in vivo. This treatment caused an energetic gap by reducing GLUT3-mediated extra glucose input and blocked the expansion process of leader cells in the tumor margin. These results indicated that GLUT3 may become a key factor correlated with tumor starvation therapy in OSCC. However, some questions still need further investigation. First, does the enhancement of glucose uptake by tumor cells via GLUT3 result in the engagement of other cells in the surrounding microenvironment? Second, whether the low expression of RRAD in tumor margin cells potentially derive from surrounding stromal cells? These questions will be further explored in our future study.

In summary, this study demonstrated that the tumor margin of OSCC showed a vigorous metabolic level. Mechanistically, the low expression of RRAD increased intracellular and nuclear Ca^2+^ concentrations and then activated the CAMKIV-CREB1 axis, which led to the upregulation of GLUT3. Furthermore, GLUT inhibitor-1 is an effective oral inhibitor that disturbs glucose metabolism and then hinders the malignant progression of OSCC. The link between RRAD and GLUT3 provides new insight into the tumor development mechanism and indicates that oral inhibitors targeting glucose metabolism are promising therapeutics for OSCC.

## Materials and methods

### Patients and tissue samples

A total of 140 OSCC tissue samples from January 2018 to December 2022 were collected to be further analyzed. Specifically, subgroups of these tissue samples were used for the analysis of spatially resolved metabolomics analysis, DSP analysis and TMA. Baseline information is listed in Supplementary Table 2. All patients enrolled signed informed consent, were pathologically diagnosed with squamous cell carcinoma and had complete clinicopathological data. The tissue samples and medical records of the enrolled patients were collected via strict procedures. This study was conducted in full accordance with ethical principles and approved by the Ethics Committee of the Beijing Stomatological Hospital, Capital Medical University.

### Spatially resolved metabolomics analysis

The embedded sample were stored at −80 °C before being sectioned. The sample were cut into consecutive 10 μm sagittal slices by a cryostat microtome (Leica CM 1950, Leica Microsystem, Wetzlar, Germany) and thaw-mounted on a positive charge desorption plate (Thermo Scientific, MA, USA). Sections were stored at −80 °C before further analysis. The samples were desiccated at −20 °C for 1 h and then at room temperature for 2 h before MSI analysis. Moreover, an adjacent slice was left for hematoxylin–eosin (H&E) staining.

This experiment was carried out with an AFADESI-MSI platform (Beijing Victor Technology Co., Ltd., Beijing, China) in tandem with a Q-Orbitrap mass spectrometer (Q Exactive, Thermo Scientific, MA, USA). Here, the solvent formula was acetonitrile (ACN)/H2O (8:2) in negative mode and ACN/H2O (8:2, 0.1%FA) in positive mode, the solvent flow rate was 5 μL/min, the transporting gas flow rate was 45 L/min, the spray voltage was set at 7 kV, and the distance between the sample surface and the sprayer was 3 mm, as was the distance from the sprayer to the ion transporting tube. The MS resolution was set at 70,000, the mass range was 70–1000 Da, the automated gain control (AGC) target was 2E6, the maximum injection time was set to 200 ms, the S-lens voltage was 55 V, and the capillary temperature was 350 °C. The MSI experiment was carried out with a constant rate of 0.2 mm/s, continuously scanning the surface of the sample section in the *x* direction and a 40-μm vertical step in the *y* direction.

The collected raw files were converted into imML format using imzMLConverter and then imported into MSiReader (an open-source interface to view and analyze high-resolving power MS imaging files on the MATLAB platform) for ion image reconstruction after background subtraction using the Cardinal software package. All MS images were normalized using total ion count normalization (TIC) in each pixel. Region-specific MS profiles were precisely extracted by matching high-spatial resolution H&E images. The discriminating endogenous molecules of different tissue microregions were screened by a supervised statistical analytical method: orthogonal partial least squares discrimination analysis (OPLS-DA). Variable importance of projection (VIP) values obtained from the OPLS-DA model were used to rank the overall contribution of each variable to group discrimination. The VIP value reflects the importance degree on the classification of sample categories with respect to the first two principal components of the OPLS-DA model, which indicates that this variable has a significant effect if the VIP is greater than 1. A two-tailed Student’s *t* test was further used to verify whether the metabolites of difference between groups were significant. Differentially abundant metabolites were selected with VIP values greater than 1.0 and *P* values less than 0.05.

In addition, for the special data structure obtained from the MSI analysis, we also performed T-distributed stochastic neighbor embedding (t-SNE) and uniform manifold approximation and projection for dimension reduction (UMAP) on the MS data in each pixel for dimensionality reduction. Spatial shrunken centroid clustering (SSCC) was applied for MSI data clustering to separate the sample based on the difference in the abundance of ions in each pixel. The ions detected by AFADESI were annotated by the pySM pipeline and an in-house SmetDB database (Lumingbio, Shanghai, China).

### DSP

Slide preparation, DSP, and subsequent data analysis were performed essentially as described by GeoMx®DSP standard operating procedures available online (https://nanostring.com). FFPE samples were dewaxed and hydrated by DSP standard. According to the DSP standard antigen repair method, an IHC pressure cooker (bioSB, CA, USA) was used for repair. Tris-EDTA buffer was used for high-temperature repair: according to the DSP standard method, proteinase K was used for digestion treatment, and formaldehyde from RNA target-exposed samples was fixed at room temperature for 5 min. The FFPE sections were incubated overnight in the hybridization furnace with CTA panel antibody and left at room temperature without light. Nuclear staining was completed by Nuclear Stain SYTO13, a standard of DSP, and detected by a machine. All slice scans were completed, and the area of interest (ROI) was selected. After exposure to UV light generated by the DSP instrument, the UV-cleaved oligo tags corresponding to discrete ROIs were released into solution and aspirated into a collection plate, followed by PCR amplification (Bio-Rad, CA, USA) with GeoMx Seq Code primers for downstream sequencing. Following pooling, purification, and quality control, the library was prepared and ready for sequencing by Illumina NovaSeq next-generation sequencing (NGS) platform (Illumina, CA, USA). After sequencing, raw reads were run through GeoMx NGS Pipelines to acquire digital count conversion (DCC) files for further processing. Differential expression analysis was performed using a DESeq2 *Q* value < 0.05 and fold change >2 or fold change <0.5 as the threshold for significant DEGs. Volcano plots were created with a *P* value set at 0.05 (dashed lines) and differential expression analysis was conducted by Mann–Whitney *U* test using R software version 4.0.2 (R Foundation for Statistical Computing, Vienna, Austria).

### mRNA sequencing

Total RNA was extracted using TRIzol reagent (Invitrogen, CA, USA) according to the manufacturer’s protocol. RNA purity and quantification were evaluated using a NanoDrop 2000 spectrophotometer (Thermo Scientific, MA, USA). RNA integrity was assessed using an Agilent 2100 Bioanalyzer (Agilent Technologies, CA, USA). Then, the libraries were constructed using the VAHTS Universal V6 RNA-seq Library Prep Kit according to the manufacturer’s instructions. Transcriptome sequencing and analysis were conducted by OE Biotech Co., Ltd. (Shanghai, China).

For RNA sequencing and DEG analysis, the libraries were sequenced on an Illumina NovaSeq 6000 platform, and 150 bp paired-end reads were generated. Raw reads in fastq format were first processed using fastp, and the low-quality reads were removed to obtain the clean reads. The clean reads were mapped to the reference genome using HISAT2FPKM, and the read counts of each gene were obtained by HTSeq-count. PCA was performed (v 3.2.0) to evaluate the biological duplication of samples.

Differential expression analysis was performed using a DESeq2 *Q* value < 0.05 and fold change >2 or fold change <0.5 as the threshold for significant DEGs. Hierarchical cluster analysis of DEGs was performed using R (v 4.0.2) to demonstrate the expression pattern of genes in different groups and samples. Based on the hypergeometric distribution, GO Pathway enrichment analyses of DEGs were performed to screen the significantly enriched terms using R (v 4.0.2). R (v 4.0.2) was used to draw the column diagram of the significantly enriched terms. The analysis used a predefined gene set, and the genes were ranked according to the degree of differential expression in the two types of samples. Then, we tested whether the predefined gene set was enriched at the top of the ranking list.

### Immunohistochemistry

IHC staining was performed to assess relative factors expression in OSCC patient tissue samples. After deparaffinization and rehydration, the tissue slides were heated in a water bath at 100 °C with citrate buffer solution (pH 6.0) for 20 min to retrieve antigen and then cooled at room temperature. The primary antibodies were incubated overnight at 4 °C in a humidified chamber and then visualized using a 3,3′-diaminobenzidine (DAB) detection kit (Dako, Glostrup, Denmark) containing goat secondary antibody molecules and DAB chromogen. Every step of the wash used phosphate-buffered saline solution (PBS) for 5 min and was repeated three times. The primary antibodies (with their dilutions reported), including RRAD (1:50, ab238584, Abcam, MA, USA), GLUT3 (1:1000, 20403-1-AP, Proteintech, Wuhan, China) and Ki-67 (1:1000, 28074-1-AP, Proteintech, Wuhan, China) were used according to the manufacturer’s instructions. The intensity of the RRAD and GLUT3 immunoreaction was scored as follows: 0 = negative, absence of stained cells; 1 = weak; 2 = moderate; and 3 = strong. The IHC staining score was calculated by multiplying the percentage of positive cells by the staining intensity. The scoring was conducted by researchers who were blinded to the clinical information of the patients.

### Multiplex immunofluorescence assay

Multiplex immunofluorescence was performed to assess RRAD, GLUT3, and Ki-67 expression in OSCC patient tissue samples. After deparaffinization and rehydration, the tissue slides were heated in a water bath at 100 °C with citrate buffer solution (pH 6.0) for 20 min to retrieve antigen and then cooled at room temperature. The primary antibodies were incubated overnight at 4 °C in a humidified chamber and then visualized using Tyramide labeling with Goat Anti-Mouse/Rabbit Multiplex IHC Detection Kit (Cat#: 18003 Zen-Bioscience Co., Ltd. Chengdu, China). The primary antibodies (with their dilutions reported), including RRAD (1:50, ab238584, Abcam, MA, USA) and GLUT3 (1:600, 20403-1-AP, Proteintech, Wuhan, China), Ki-67 (1:400, 28074-1-AP, Proteintech, Wuhan, China) were used according to the manufacturer’s instructions.

### Cell culture

CAL27, HN4, and HN30 cell lines were used in this study. Three cell lines were recently authenticated by STR profiling, and no mycoplasma contamination was detected. Cell lines were cultured in Dulbecco’s modified Eagle’s medium (Gibco, CA, USA) with 10% fetal bovine serum and 1% penicillin–streptomycin. Cells were cultured in a standard humidified atmosphere of 5% CO_2_ at 37 °C.

### Cell transfection

SiRNA for both RRAD and CAMKIV (Syngenbio Co., Ltd., Beijing, China), as well as scramble sequence, were transiently transfected into HN4 and CAL27 cells using Lipofectamine™ 3000 (Invitrogen, CA, USA) according to the manufacturer’s instructions. The subsequent treatments and experiments were performed 24 h after transfection. The sequences of the RRAD siRNAs were as follows: #1, 5′-GCGAGAGAGCCUUGGCAAATT-3′; #2, 5′-CCAUCAUCCUCGUGGGCAATT-3′; and #3, 5′-CCAUUGUAGUGGACGGAGATT-3′. siRNA #1 was chosen because it had the highest silencing efficiency. The sequences of the CAMKIV siRNAs were as follows: #1, 5′-AGUUAAAGGUGCAGAUAUATT-3′; #2, 5′-CAUCUUACUUUGUGGAUUUTT-3′; and #3, 5′-CCAAAGAAACGGCUGACUATT-3′. siRNA #1 was chosen because it had the highest silencing efficiency.

In the in vivo experiments, the RRAD knockdown lentivirus was utilized for transfection of HN4 cell lines for subsequent endeavors. The RRAD knockdown lentivirus was custom-made by Syngenbio Co., Ltd., Beijing, China. The plasmid sequence of this knockdown lentivirus vector is pLV-hU6-shRNA-CMV-Puro. The lentiviral plasmid carries the shRNA sequence of sh-RRAD UCUCCGUCCACUACAAUGGTT. The lentiviral transfections were implemented once the HN4 cells in the six-well plate attained a 40% confluence. After 48 h of viral infection, various concentrations of puromycin (2.5 mg/mL for HN4) were employed to select for stable expressing cell lines. The success of transfection was confirmed through Western blot and RT-PCR analysis.

### Real-time PCR

For determination of the expression levels of RRAD, SLC2A1, SLC2A2, SLC2A3, and SCL2A4, RT-PCR was performed in CAL27 and HN4 cell lines. Cells were extracted with TRIzol. Complementary DNA (cDNA) was synthesized from total RNA by M‐MLV reverse transcriptase (TaKaRa, Tokyo, Japan) after extraction and centrifugation. Quantitative gene analysis was performed using SYBR Premix Ex Taq II (TaKaRa, Tokyo, Japan) and an RT‐PCR analyzer (Bio-Rad, CA, USA). Relative gene quantitation was assessed by the 2‐ΔΔCT method. Primers are listed as follows.

RRAD forward: 5′-GCAGGGCACACCTATGATCG-3′ and reverse: 5′-GCTCCCAAATGTCGTAGACCA-3′;

SLC2A1 forward: 5′-GGCCAAGAGTGTGCTAAAGAA-3′ and reverse: 5′- ACAGCGTTGATGCCAGACAG-3′;

SLC2A2 forward: 5′-GCTGCTCAACTAATCACCATGC-3′ and reverse: 5′-TGGTCCCAATTTTGAAAACCCC-3′;

SLC2A3 forward: 5′-TGGTCCCAATTTTGAAAACCCC-3′ and reverse: 5′-GCACTTTGTAGGATAGCAGGAAG-3′;

SLC2A4 forward: 5′-TGGGCGGCATGATTTCCTC-3′ and reverse: 5′-GCCAGGACATTGTTGACCAG-3′.

### Western blot analysis

Western blotting was performed as follows. Briefly, cells were washed three times with ice-cold PBS and then lysed on ice in SDS lysis buffer (50 mM Tris, 1% SDS, protease inhibitors and phosphatase inhibitors). The protein concentration was determined with Coomassie Brilliant Blue (Bio-Rad, CA, USA). A total of 25 μg of protein lysates was separated by 10% sodium dodecyl sulfate-polyacrylamide gel electrophoresis (SDS-PAGE) and transferred onto polyvinylidene difluoride (PVDF) membranes (Millipore, MA, USA) using a wet transfer system (Bio-Rad, CA, USA). Membranes were blocked with 5% w/v skim milk, incubated overnight in primary antibody with gentle shaking at 4 °C, and then incubated with secondary antibody for 1 h at room temperature. Detection was performed with a ChemiDoc MP imaging system (Bio-Rad, CA, USA).

The antibodies used in this study were RRAD (ab177151, Abcam, MA, USA), CAMKIV (4032 Cell Signaling Technology, MA, USA), p-CAMKIV (ab195000, Abcam, MA, USA), CREB1 (48H2, Cell Signaling Technology, MA, USA), p-CREB1 (87G3, Cell Signaling Technology, MA, USA), GLUT1 (bs-0472R, Bioss, Beijing China), GLUT2 (bs-0351R, Bioss, Beijing, China), GLUT3 (20403-1-AP, Proteintech, Wuhan, China), and GLUT4 (bs-0384R, Bioss, Beijing, China). GAPDH (60004-1-Ig, Proteintech, Wuhan, China) and histone H3 (ab1791, Abcam, MA, USA) were used. GAPDH and histone H3 were used as internal controls. All western blots were repeated three times with separate cell lysates. Densitometric values for all western blot experiments were analyzed using the software ImageJ and the statistical results were uploaded as a Supplementary file.

### Measurement of cellular Ca^2+^ concentration and Ca^2+^ distribution

Cell lines were cultured with the indicated conditions. Thereafter, the cells were loaded with Fluo-4 AM (Beyotime, Shanghai, China) for 30 min at 37 °C to observe Ca^2+^ distribution and Ca^2+^ concentration. Cellular Ca^2+^ showed green fluorescence. Evaluation of the cellular Ca^2+^ concentration was based on the fluorescence intensity detected by the Multi-Mode Detection Platform (SpectraMax Paradigm, Molecular Devices, Co., CA, USA). Images were captured with an Ax10 Axio fluorescence microscope (Zeiss, Oberkochen, German).

As for the tumor cells separated from fresh tissue, we referred to the experimental method published in previous research [41]. Briefly, fresh primary lesion biopsies were obtained during surgery in patients with advanced OSCC. They were immediately stored in DMEM at 4 °C for ~30–50 min prior to pathological characterization. After immediate H&E staining of frozen samples during surgery to ensure that samples simultaneously contained tumor margin, tumor center, and normal region. The tumor margin and tumor center were separated under the guidance of pathology experts. The separate regions were then digested for the preparation of single cell suspensions and inoculation into six-well plates for primary culture. Over the next few days, gradient centrifugation, digestion elimination, and repeated adhesion were used to remove other types of cells. The Fluo-4 AM fluorescent labeling of Ca^2+^ was used to determine the concentration of Ca^2+^, as mentioned above.

### Glucose uptake assay

A total of 5 × 10^3^ cells were cultured in 96-well plates containing glucose-free DMEM (Thermo Scientific, MA, USA) with 10% fetal bovine serum and 1% penicillin–streptomycin, transferred to a CO_2_ incubator set at 37 °C and 5% CO_2_, and incubated for 12 h. Following the manufacturer’s instructions, 2-(N-(7-nitrobenz-2-oxa-1,3-diazol-4-yl)amino)-2-deoxyglucose (2-NBDG) (Thermo Fisher Scientific, MA, USA) was used to measure glucose uptake on the basis of fluorescence intensity detected by a Multi-Mode Detection Platform (SpectraMax Paradigm, Molecular Devices Co., CA, USA).

### Lactate product assay

A total of 1 × 10^4^ cells were cultured in 12-well plates in the indicated conditions for 24 h. According to the manufacturer’s instructions, spent media were collected to measure lactate with a Lactate Assay Kit (Dojindo, Kumamoto, Japan).

### Measurement of the ADP:ATP ratio

The cellular energy status was assessed by measuring the ADP:ATP ratio, and ADP and ATP levels in the samples were determined using the ADP: ATP Ratio Assay Kit-Luminescence (Dojindo, Kumamoto, Japan). All samples were run as duplicates in three independent experiments. ADP:ATP ratios were calculated based on the manufacturer´s recommendation.

### Metabolic flux assay

The ECAR was analyzed on an XF96 Extracellular Flux Analyzer (Agilent Technologies, CA, USA). Cells were seeded in XFe 96-well microplates (30,000 cells/well) for 12 h, washed, and incubated in XF RPMI medium at 37 °C for 1 h. The ECAR was measured in response to 0.5 μM rotenone and 50 mM 2-deoxy-D-glucose in real-time conditions.

### Nuclear isolation

CAL27 cells or HN4 cells were collected and lysed with a Nuclear and Cytoplasmic Protein Extraction Kit (Beyotime, Shanghai, China) with 1 mM PMSF. The cell suspension was left on ice for 15 min, and then, the homogenates were centrifuged (16,000×*g*) for 5 min at 4 °C. The obtained supernatant was used to extract cytoplasmic protein. The remaining deposit was further centrifuged (16,000×*g*) for 10 min and collected as a nuclear protein.

### EdU assay

The treated HN4 and CAL27 cells were incubated with 10 μM EdU (Beyotime, Shanghai, China) for 2 h in six-well plates. After fixation with paraformaldehyde and washing, the cells were treated with 0.5 ml of Click Additive Solution for 30 min. Then, the DNA of the cells was stained with Hoechst for 10 min and visualized under an Ax10 Axio fluorescence microscope (Zeiss, Oberkochen, Germany).

### Chromatin immunoprecipitation (ChIP) assay

ChIP assays were performed on HN4 and CAL27 cells using a Chromatin Immunoprecipitation (ChIP) Assay Kit (P2078, Beyotime, Shanghai, China). IgG was used as the negative control. A CREB1 antibody (48H2, Cell Signaling Technology, MA, USA) was used to pull down the target genes in the promoter regions. ChIP DNAs were analyzed using qPCR and were normalized to the input data.

### Cellular proliferation, migration, and invasion assays

Cell counting kit (CCK-8; Dojindo, Kumamoto, Japan) assays were used to detect cell proliferation. Migration was examined by both a wound-healing assay and a Transwell assay (uncoated insert). A Transwell assay (Matrigel-coated insert) was used for invasion monitoring. Cell migration and invasion assays were performed using a Transwell technique with uncoated polycarbonate inserts (Millipore, Darmstadt, Germany) or BioCoat™ inserts (BD Biosciences, NJ, USA). Medium without FBS (2 × 10^4^ cells/200 μL) was added to the upper portion of a migration (uncoated insert) or invasion (Matrigel-coated insert) chamber, with 600 μL of DMEM containing 10% FBS added to the lower chamber. All experiments were performed three times, and the cell numbers were counted at least three times to calculate an average.

### Animal studies

Six-week-old BALB female nude mice purchased from SPF Biotechnology Co., Ltd. (Beijing, China), were bred in SPF facilities. The mice were randomly divided into groups (*n* = 5), and the animal study was conducted by the researchers, who were blinded to the treatment. A total of 1 × 10^6^ HN4 cells were injected into the left or right flanks of mice for tumorigenicity evaluation. The GLUT inhibitor-1 (HY-139605, MedChem Express, NJ, USA) was mixed into drinking water once a day in the first two weeks (30 mg/kg), and DMSO was used as the solvent control. Tumor volume was measured once a week. The weight and volume of the tumors were finally measured after the mice were sacrificed at the endpoint.

Tumor tissues were fixed and stained before microscopic analysis. The in vivo studies were approved by the Animal Care and Use Committee of Beijing Stomatological Hospital, Capital Medical University.

### Statistical analysis

The data were analyzed by SPSS 22.0 for Windows (SPSS, Inc., IL, USA), and R software version 4.0.2 (R Foundation for Statistical Computing, Vienna, Austria) was used to build a predictive model. All the data were plotted by GraphPad Prism version 9 (GraphPad Software, CA, USA). Student’s *t* test and one-way ANOVA were performed to assess the statistical significance of differences. Survival analysis was conducted using the Kaplan–Meier method and log-rank test. *P* < 0.05 was considered statistically significant (*P < 0.05, **P < 0.01, and ***P < 0.001). All values are expressed as the means ± standard errors.

### Supplementary information

Supplementary materials

Original western blot

## Data Availability

The data in this study are available from the corresponding authors upon reasonable request.
