# Supplementary material for: The enhanced energy metabolism in the tumor margin mediated by RRAD promotes the progression of oral squamous cell carcinoma
Source: Cell Death Dis. 2024 May 29;15(5):376. doi: 10.1038/s41419-024-06759-7 (PMC11137138; doi:10.1038/s41419-024-06759-7)
Supplement: Supplementary file 1 — Supplementary materials [file 41419_2024_6759_MOESM1_ESM.pdf]

Supplementary materials

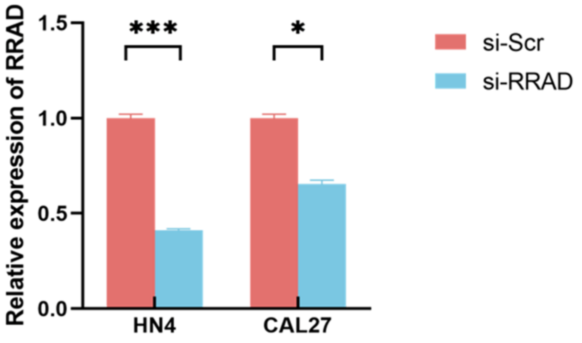

Supplementary Figure 1. Statistical analysis of Western blot in Figure 2:

The statistical analysis of the expression of RRAD after RRAD-specific siRNA transfection for 48 h in HN4 and CAL27 cells. *P<0.05, ***P<0.001.

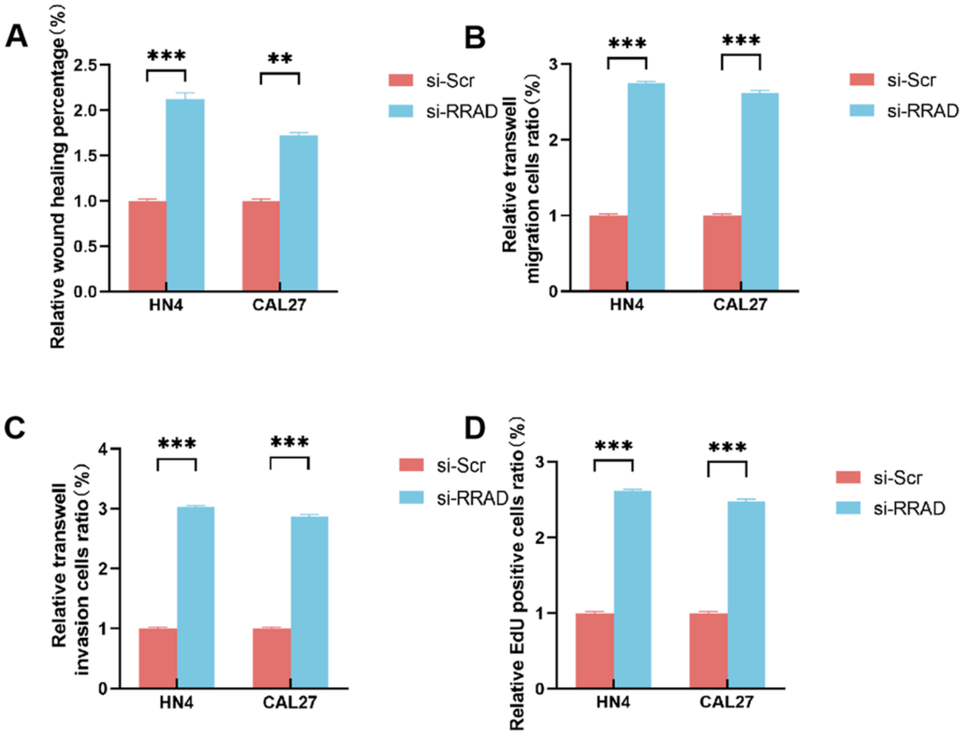

Supplementary Figure 2. Quantitative statistical analysis of Cell phenotype assays in Figure 2:

Quantitative statistical analysis of wound healing (A), Transwell (B-C) and EdU (D) assay after RRAD-specific siRNA transfection in HN4 and CAL27 cells. **P<0.01, ***P<0.001.

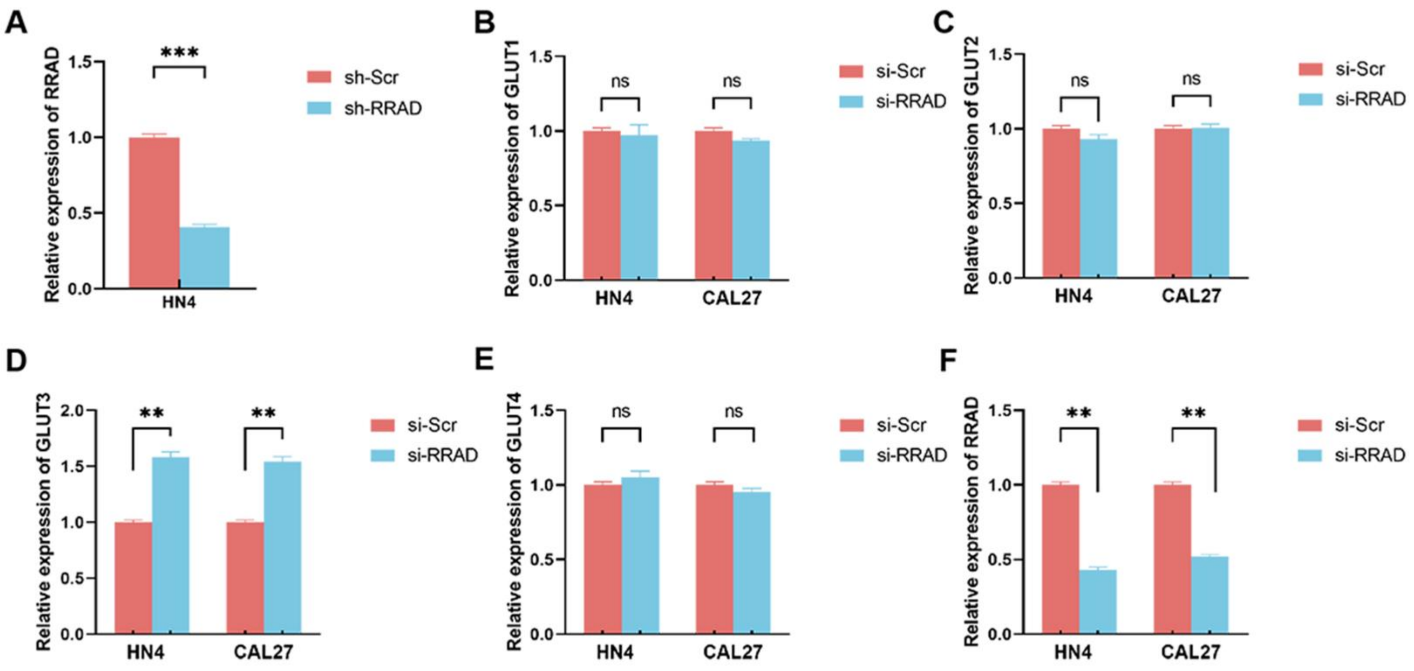

Supplementary Figure 3. Statistical analysis of Western blot in Figure 3:

The statistical analysis of the expression of RRAD after RRAD specific shRNA transfection in HN4 cells(A). The statistical analysis of the expression of GLUT1 (B), GLUT2 (C), GLUT3 (D), GLUT4 (E), RRAD (F) after RRAD-specific siRNA transfection in HN4 and CAL27 cells. **P<0.01, ***P<0.001.

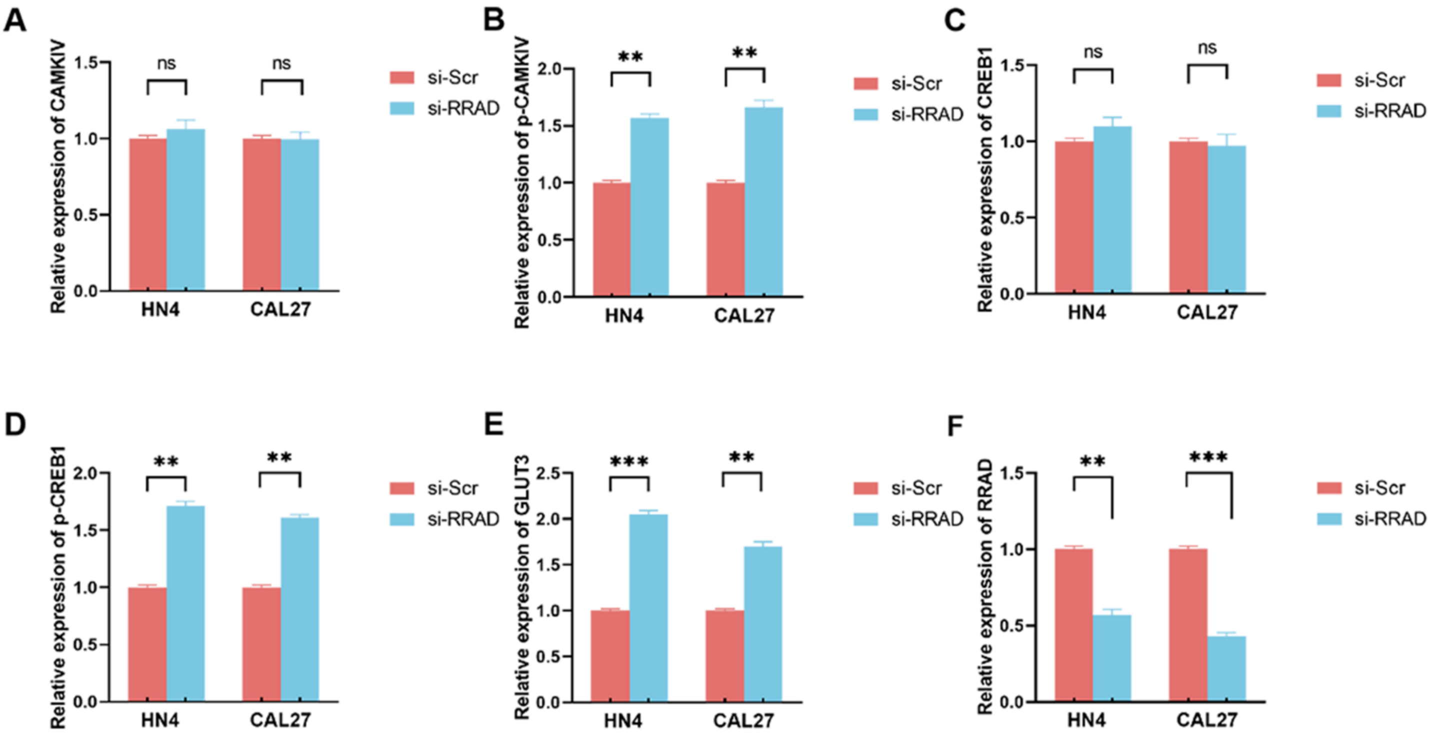

Supplementary Figure 4. Statistical analysis of Western blot in Figure 4D:

The statistical analysis of the expression of CAMKIV (A), p-CAMKIV (B), CREB1 (C), p-CREB1 (D) in nucleus and GLUT3 (E), RRAD (F) in cytoplasm after RRAD-specific siRNA transfection in HN4 and CAL27 cells. **P<0.01, ***P<0.001.

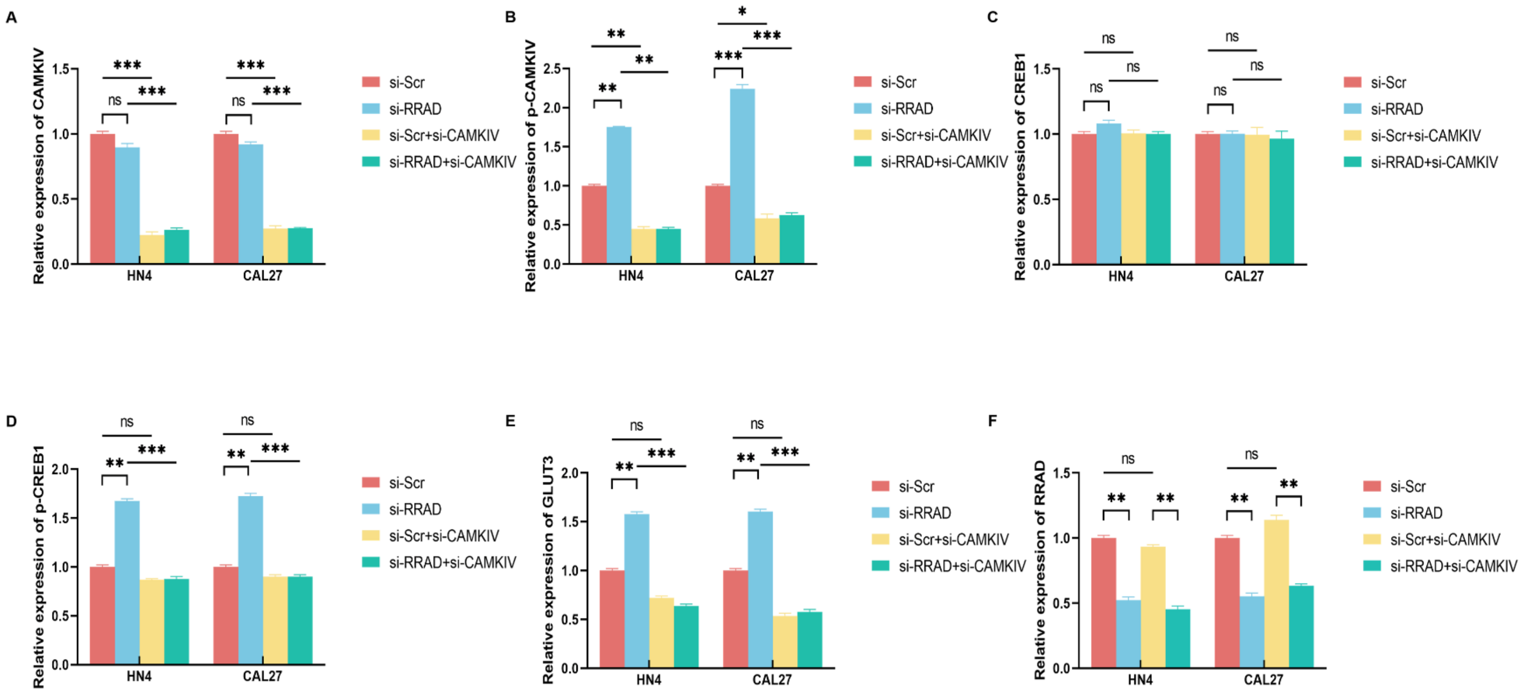

Supplementary Figure 5. Statistical analysis of Western blot in Figure 4E:

The statistical analysis of the expression of CAMKIV (A), p-CAMKIV (B), CREB1 (C), p-CREB1 (D) in nucleus and GLUT3 (E), RRAD (F) in cytoplasm after RRAD-specific siRNA and CAMKIV specific siRNA transfection in HN4 and CAL27 cells. *P<0.05, **P<0.01, ***P<0.001.

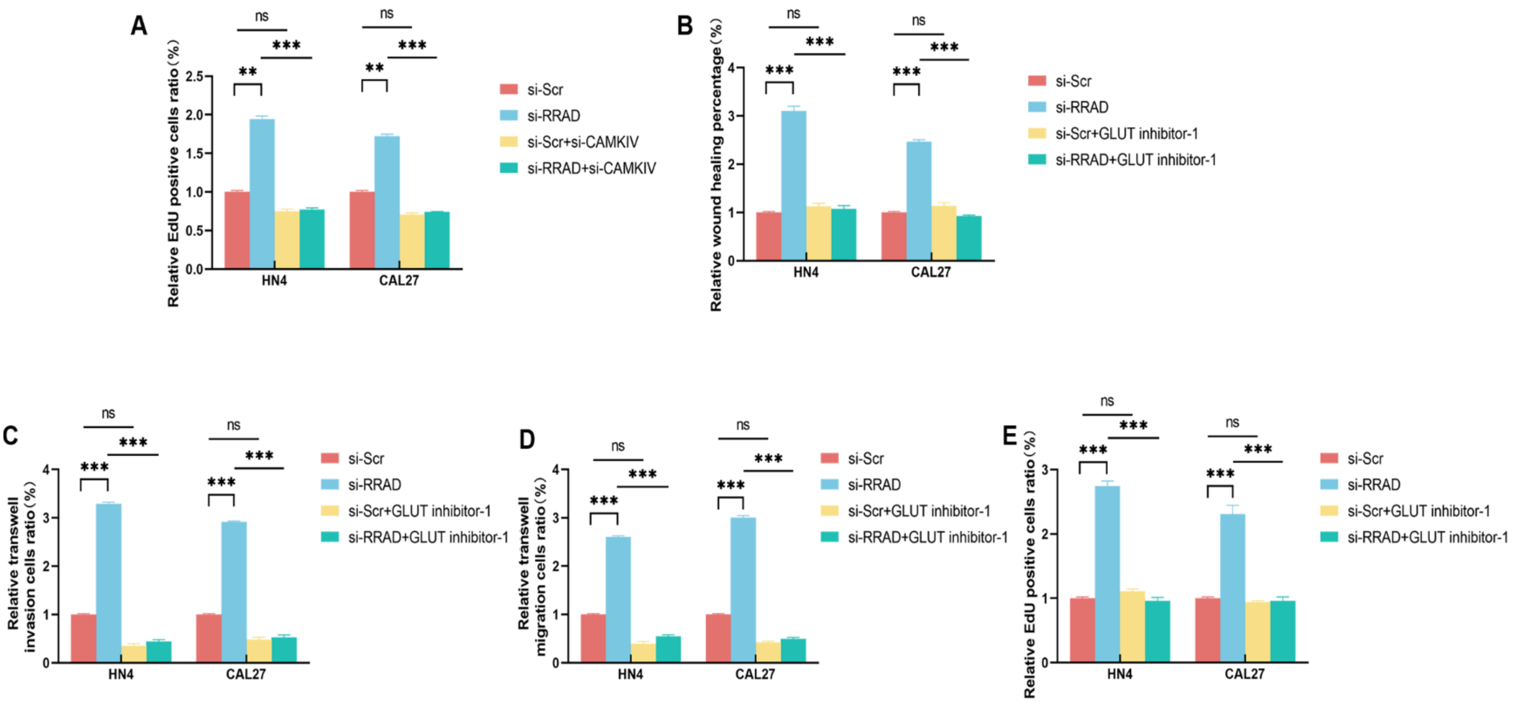

Supplementary Figure 6. Quantitative statistical analysis of Cell phenotype assays in Figure 4 and Figure 5:

Quantitative statistical analysis of EdU (A) assay after RRAD-specific siRNA and CAMKIV specific siRNA transfection in HN4 and CAL27 cells. Quantitative statistical analysis of wound healing (B), Transwell (C-D) and EdU (E) assay after RRAD-specific siRNA transfection and treated with GLUT inhibitor-1 in HN4 and CAL27 cells. **P<0.01, ***P<0.001.

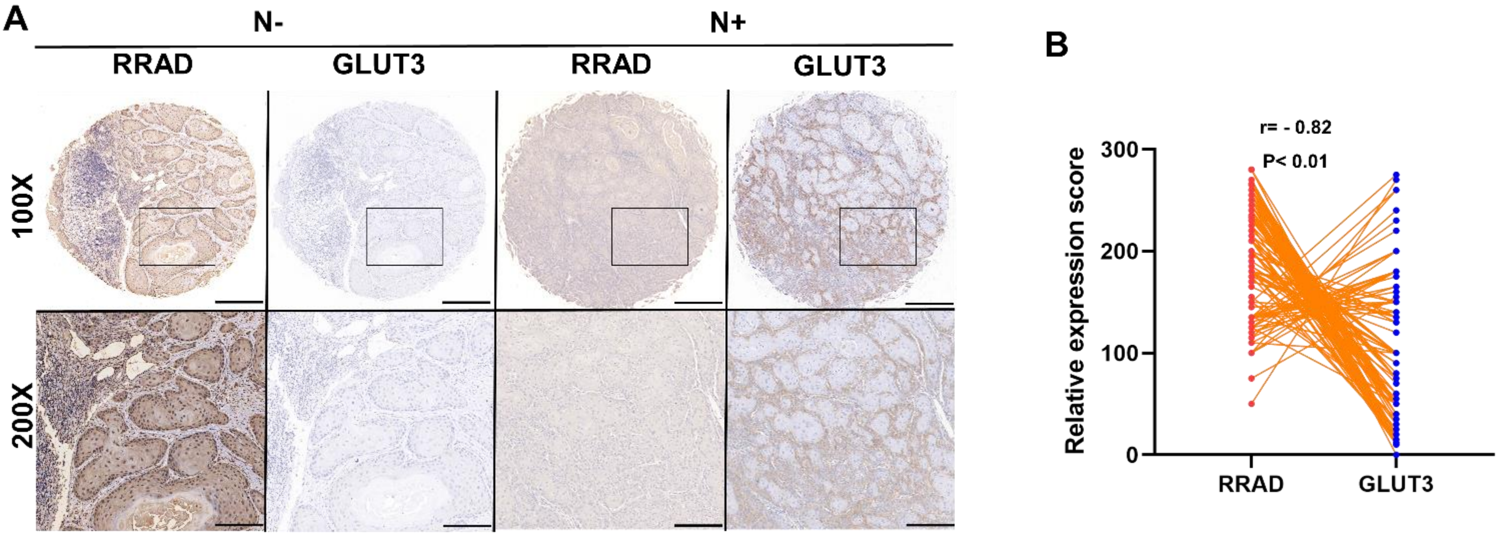

Supplementary Figure 7. Representative IHC images and correlation analysis of RRAD and GLUT3 based on OSCC tumor tissues:

Representative IHC images and correlation analysis of RRAD and GLUT3 based on 135 OSCC tumor tissues (A). Relative expression score of RRAD and GLUT3 (r=-0.82, P<0.01) (B). Scale bar: 500 µm and 200 µm.

Supplementary Table 1： The DEGs of tumor margin identified through spatial transcriptome profiling based on the NanoString DSP instrument

Gene	Regulation	Log2FC	Pvalue	Gene	Regulation	Log2FC	Pvalue
**RSAD2**	DOWN	-1.897067778	0.011176274	**IFI6**	DOWN	-2.261511472	0.006596096
**BIRC3**	DOWN	-2.459102989	0.000449423	**KIR3DL3**	DOWN	-2.10795681	1.14729E-14
**NPM1**	DOWN	-1.159029238	0.008415226	**IFI27**	DOWN	-1.865199638	0.002274874
**BST2**	DOWN	-1.162096726	0.018831095	**ISG15**	DOWN	-2.199628567	0.038935008
**DDIT4**	DOWN	-2.709925296	3.94609E-09	**CD24**	DOWN	-1.564951665	0.000354773
**HLA-C**	DOWN	-1.643519085	0.003741791	**RRAD**	DOWN	-1.201314336	0.017187592
**LY6E**	DOWN	-2.665374021	0.002702656	**SAMD9**	DOWN	-1.200143575	0.006759922
**APOE**	DOWN	-1.346928162	2.49232E-11	**OAS1**	DOWN	-1.131303746	0.019537259
**DSC3**	DOWN	-1.146072611	0.035249348	**MEF2C**	UP	1.090234686	1.59436E-06
**FSTL3**	DOWN	-1.063285858	0.000571147	**COL1A1**	UP	2.321196301	2.95961E-06
**MICA**	DOWN	-1.391263128	6.59436E-14	**CXCL9**	UP	1.278082315	0.000140959
**HSPB1**	DOWN	-2.255083293	7.18393E-06	**COL6A3**	UP	1.666279489	9.11262E-09
**TACSTD2**	DOWN	-1.210539994	0.01379575	**COL3A1**	UP	2.30795335	1.42734E-07
**DUSP1**	DOWN	-1.365410098	0.002934975	**COL1A2**	UP	2.087052588	2.07687E-07
**RHOB**	DOWN	-1.696166811	0.000888708	**TPM1**	UP	1.42714187	0.000608994
**SLC16A1**	DOWN	-1.000691082	0.018964185	**SFRP2**	UP	2.005237815	5.06652E-29
**HSPA1A**	DOWN	-1.386923149	0.008010901	**KRT19**	UP	1.218928613	2.04E-06
**ADM**	DOWN	-1.535399265	2.38292E-05	**THBS4**	UP	2.611492374	5.2969E-23
**IL20RB**	DOWN	-1.086150788	0.027025163	**A2M**	UP	1.299355067	4.21554E-07
**ERO1A**	DOWN	-1.274335302	0.000931775	**FLNC**	UP	1.458146298	2.56226E-11

Supplementary Table 2： Baseline information of 140 patients

Baseline information of 135 patients for RRAD and GLUT3 IHC staining

Variable	Patients (n=135)	
	No.	%
**Age: mean±SD**	64.15±12.81	
**Gender**		
Male	67	49.6
Female	68	50.4
**Sites**		
Tongue	58	42.9
Lower gingiva	20	15.4
Buccal	32	23.6
Floor of the mouth	6	4.3
Upper gingiva	16	11.6
Hard palate	3	2.2
**Pathological**		
**T classification**		
T1	10	7.4
T2	41	30.4
T3	16	11.8
T4	68	50.4
**Pathological grade**		
Well	66	48.8
Moderate	48	35.6
Poor	21	15.6
**Smoking history**		
Smoker	88	65.2
Non-smoker	47	34.8
**Alcohol history**		
Drinker	98	72.6
Non-drinker	37	27.4
**lymph node metastases**		
Absence	50	37.1
Presence	85	62.9

Baseline information of 1 patient for spatially resolved metabolomics analysis

Variable	
**Age:**	64
**Gender**	Male
**Sites**	Floor of the mouth
**Pathological T classification**	T2
**Pathological grade**	Moderate
**Smoking history**	Smoker
**Alcohol history**	Drinker
**lymph node metastases**	Presence

Baseline information of 4 patients for spatial transcriptome profiling based on the NanoString DSP instrument

Variable	Patients (n=4)	
	No.	%
**Age: mean±SD**	59±5.72	
**Gender**		
Male	2	50.0
Female	2	50.0
**Sites**		
Tongue	4	100
Lower gingiva	0	0.0
Buccal	0	0.0
Floor of the mouth	0	0.0
Upper gingiva	0	0.0
Hard palate	0	0.0
**Pathological**		
**T classification**		
T1	0	0.0
T2	2	50.0
T3	1	25.0
T4	1	25.0
**Pathological grade**		
Well	4	100
Moderate	0	0.0
Poor	0	0.0
**Smoking history**		
Smoker	1	25.0
Non-smoker	3	75.0
**Alcohol history**		
Drinker	1	25.0
Non-drinker	3	75.0
**lymph node metastases**		
Absence	2	50.0
Presence	2	50.0
