# Supplementary figures and images for: The enhanced energy metabolism in the tumor margin mediated by RRAD promotes the progression of oral squamous cell carcinoma
Source: Cell Death Dis. 2024 May 29;15(5):376. doi: 10.1038/s41419-024-06759-7 (PMC11137138; doi:10.1038/s41419-024-06759-7)

Figure 2B

RRAD

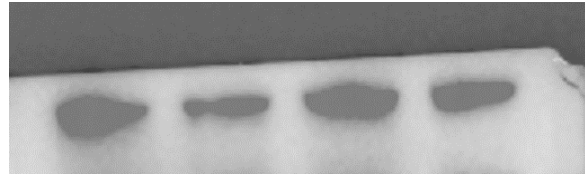

GAPDH

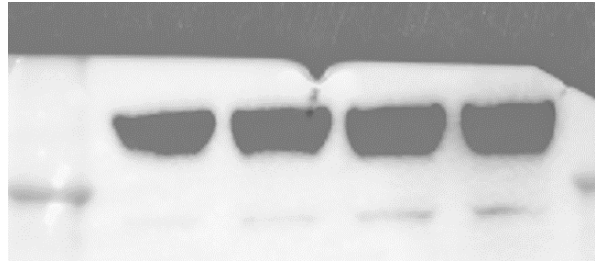

Figure 3B

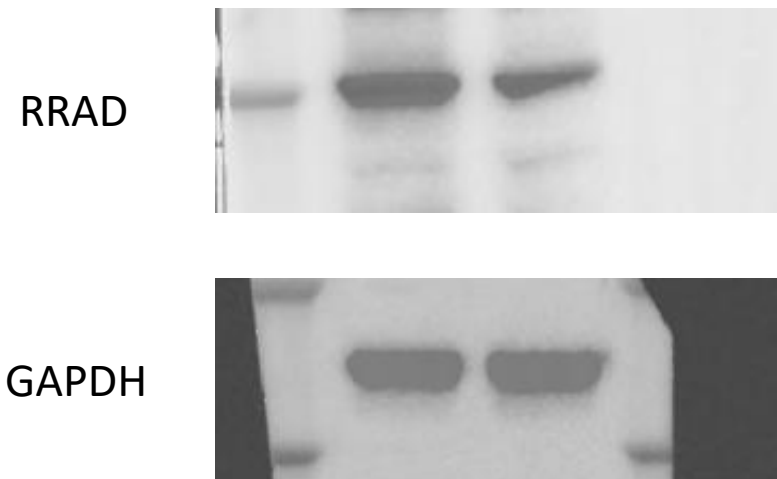

Figure3K

GLUT1

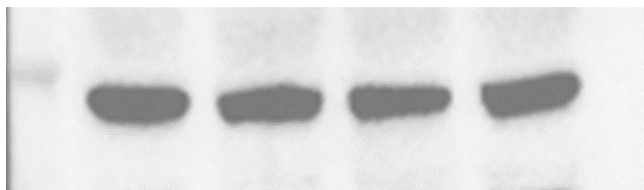

GLUT2

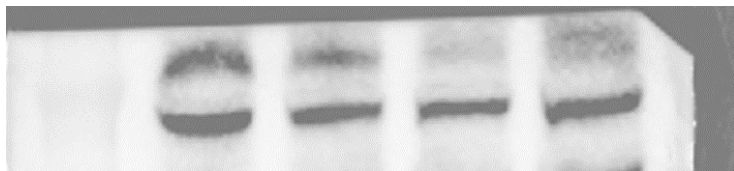

GLUT3

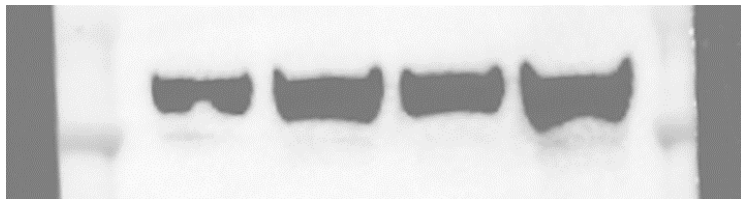

GLUT4

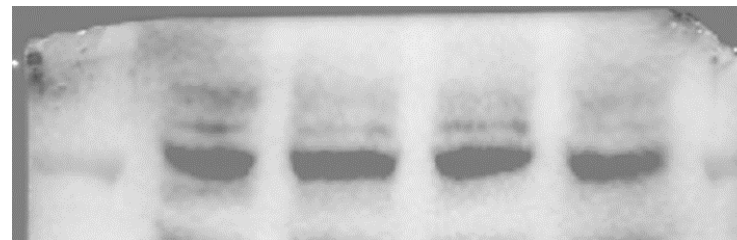

RRAD

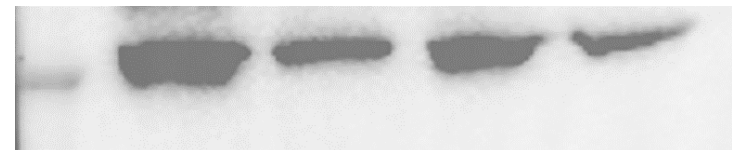

GAPDH

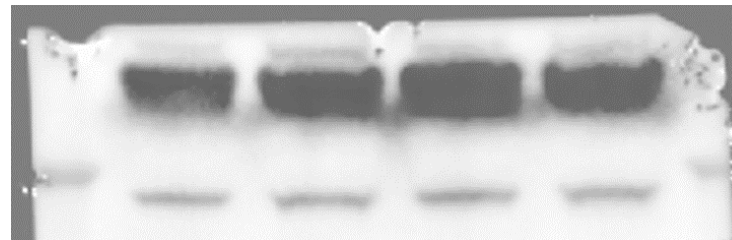

Figure4D

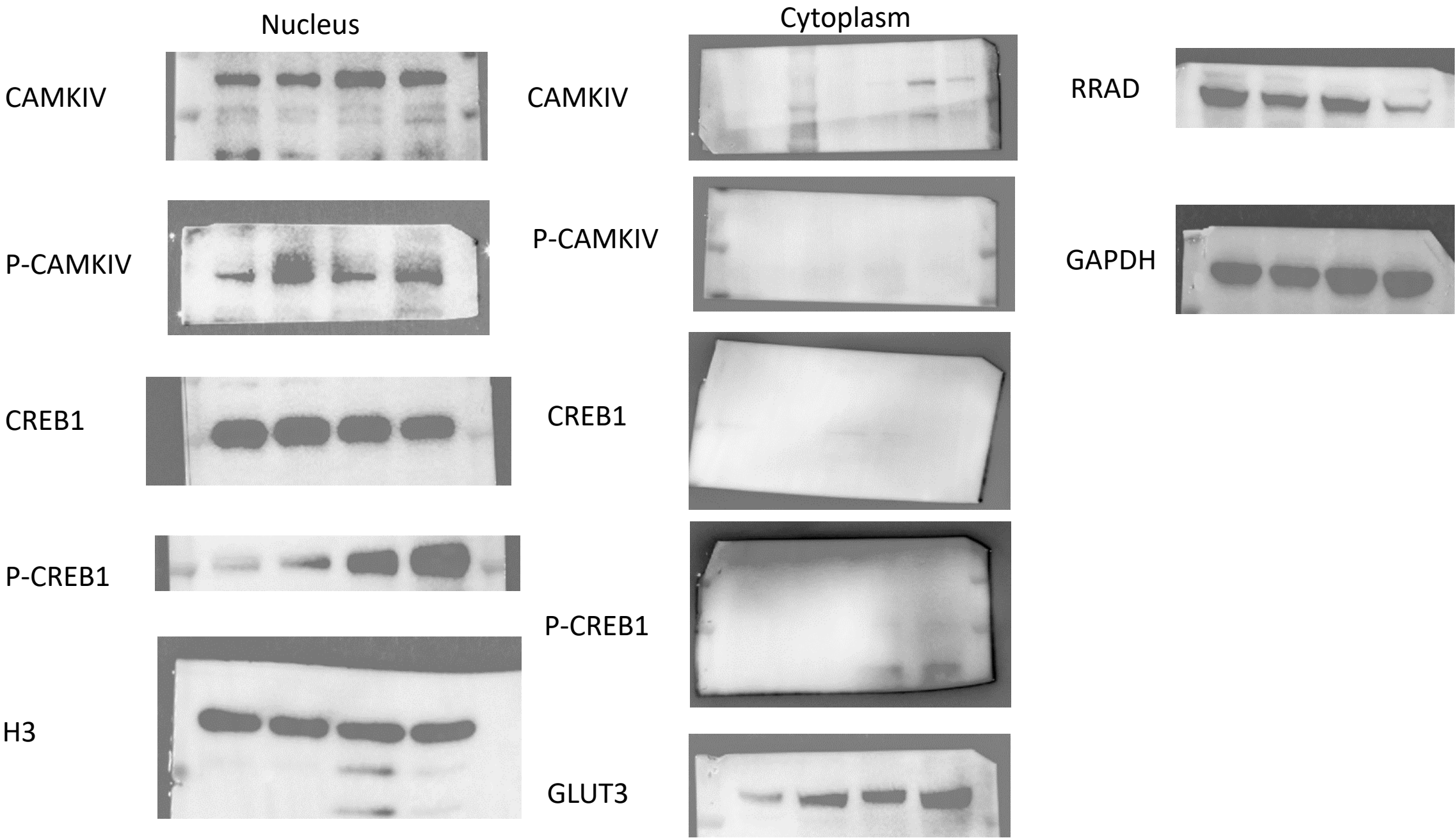

Figure4E

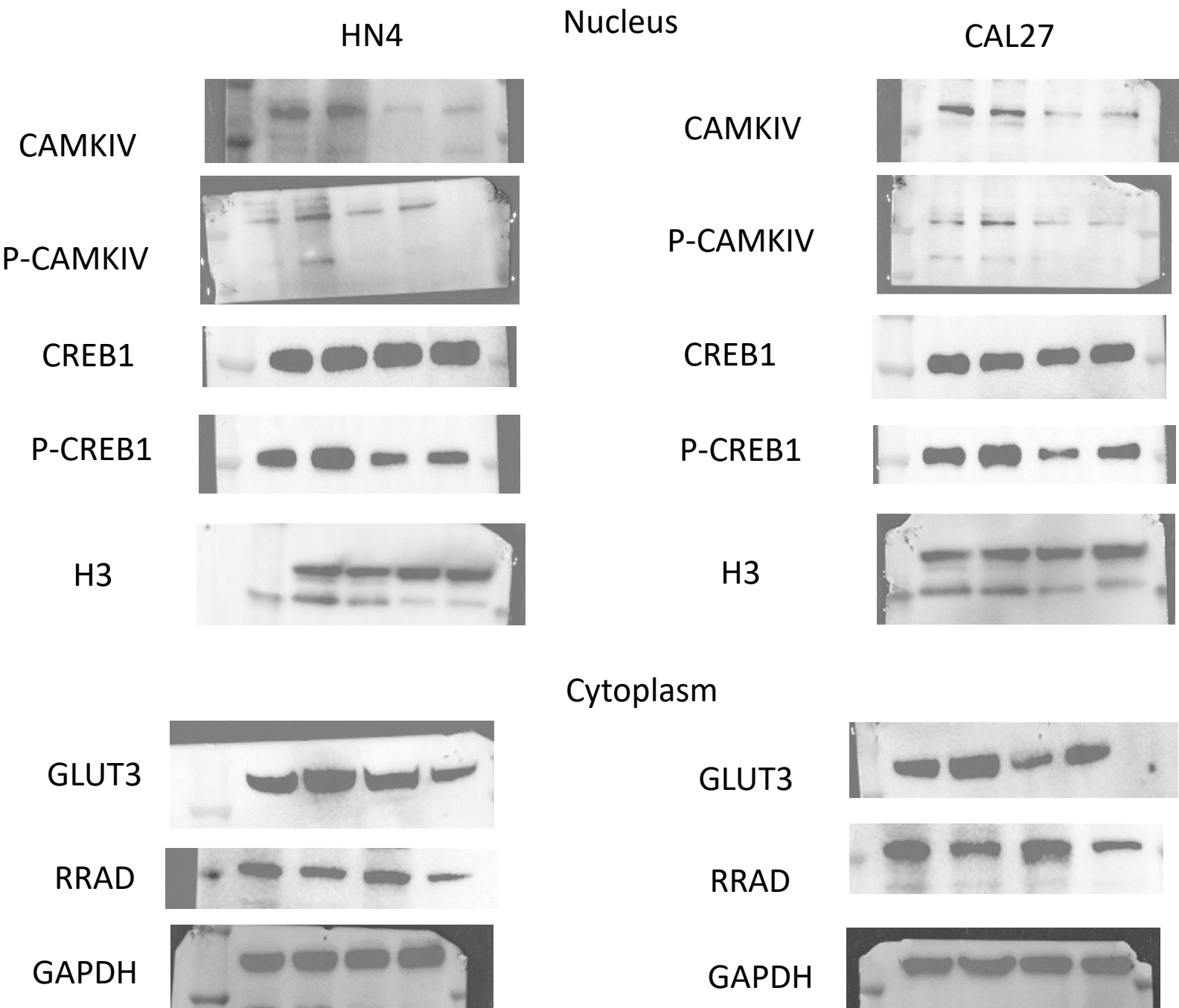

Supplement: Supplementary file 2 — Original western blot [file 41419_2024_6759_MOESM2_ESM.pdf]
